# Cysteine protease inhibitor 1 promotes metastasis by mediating an oxidative phosphorylation/MEK/ERK axis in esophageal squamous carcinoma cancer

**DOI:** 10.1038/s41598-024-55544-1

**Published:** 2024-02-29

**Authors:** Liangming Zhang, Xiongfeng Chen, Jianwei Wang, Meihong Chen, Juan Chen, Wanzhen Zhuang, Yu Xia, Zhixin Huang, Yue Zheng, Yi Huang

**Affiliations:** 1 Shengli Clinical Medical College, Fujian Medical University, No.134 East Street, Fuzhou, 350001 Fujian Province China; 2 Department of Clinical Laboratory, Fujian Provincial Hospital South Branch, Fuzhou, 350008 Fujian China; 3 Department of Scientific Research, Fujian Provincial Hospital, Fuzhou, 350001 Fujian China; 4 Clinical Laboratory Department of Fuding Hospital, Fujian University of Traditional Chinese Medicine, Fuding, 355200 Fujian China; 5 Department of Clinical Laboratory, Fujian Provincial Hospital, Fuzhou, 350001 Fujian China; 6 Integrated Chinese and Western Medicine College, Fujian University of Traditional Chinese Medicine, Fuzhou, 350000 Fujian China; 7 Central Laboratory, Center for Experimental Research in Clinical Medicine, Fujian Provincial Hospital, Fuzhou, 350001 Fujian China; 8 Fujian Provincial Key Laboratory of Critical Care Medicine, Fujian Provincial Key Laboratory of Cardiovascular Disease, Fuzhou, 350001 Fujian China

**Keywords:** CST1, Esophageal squamous cell carcinoma, Oxidative phosphorylation, MEK/ERK pathway, Cancer, Metastasis, Tumour biomarkers

## Abstract

Cysteine protease inhibitor 1 (CST1) is a cystatin superfamily protein that inhibits cysteine protease activity and is reported to be involved in the development of many malignancies. Mitochondrial oxidative phosphorylation (OXPHOS) also plays an important role in cancer cell growth regulation. However, the relationship and roles of CST1 and OXPHOS in esophageal squamous cell carcinoma (ESCC) remains unclear. In our pilot study, CST1 was shown the potential of promoting ESCC migration and invasion by the activation of MEK/ERK pathway. Transcriptome sequencing analysis revealed that CST1 is closely associated with OXPHOS. Based on a real-time ATP rate assay, mitochondrial complex I enzyme activity assay, immunofluorescence, co-immunoprecipitation, and addition of the OXPHOS inhibitor Rotenone and MEK/ERK inhibitor PD98059, we determined that CST1 affects mitochondrial complex I enzyme activity by interacting with the GRIM19 protein to elevate OXPHOS levels, and a reciprocal regulatory relationship exists between OXPHOS and the MEK/ERK pathway in ESCC cells. Finally, an in vivo study demonstrated the potential of CST1 in ESCC metastasis through regulation of the OXPHOS and MEK/ERK pathways. This study is the first to reveal the oncogenic role of CST1 in ESCC development by enhancing mitochondrial respiratory chain complex I activity to activate the OXPHOS/MEK/ERK axis, and then promote ESCC metastasis, suggesting that CST1/OXPHOS is a promising target for ESCC treatment.

## Introduction

Esophageal cancer is the eighth most frequent cancer and the sixth leading cause of cancer-related deaths worldwide, accounting for more than 500,000 deaths annually^1^, which can be classified as esophageal squamous cell carcinoma (ESCC) and esophageal adenocarcinoma (EAC)^2^. Over the last few years, ESCC has been reported to have a noticeably higher incidence rate than that of EAC, as well as a dismal five-year survival rate of 5–20%^3,4^. Unfortunately, China presents the highest incidence of ESCC, accounting for more than 50% of new cases worldwide^5^. Therefore, exploring the pathogenesis of ESCC and identifying early screening indicators are crucial.

Oxidative phosphorylation (OXPHOS) and glycolysis are the two critical energy metabolic pathways in mammalian cells^6–10^. OXPHOS occurs mainly through the cellular mitochondrial respiratory chain complex pathway to synthesize large amounts of ATP to meet the daily needs of the cells^11–13^. There is growing evidence that certain types of malignancies depend on OXPHOS for further progression^14,15^. For example, OXPHOS can activate oncogenic pathways such as the Erk1/2 pathway and Akt pathway, to promote cancer cell proliferation and metastasis^16,17^. In addition, OXPHOS inhibitors have been reported to effectively target certain cancers, including thyroid and endometrial cancers, etc^18,19^. Thus, targeting OXPHOS is being a promising strategy for the treatment of certain malignancies.

Cysteine proteinase inhibitor 1 (CST1) is a secreted protein that belongs to the cysteine proteinase inhibitor (CST) type 2 subfamily^20–23^. Aberrant CST1 expression has been reported to be associated with the development of some malignancies, such as breast, lung, liver and gastric cancers^24–28^. While other CST family proteins have not been further investigated in relation to tumorigenesis and progression. Encouragingly, our pilot study uncovered the aberrantly high expression of CST1 in both the serum and cancerous tissues of ESCC patients^29^, and the effect of the miR-942-5p/CST1 axis on the migration and invasion of ESCC cells via the MEK/ERK signaling pathway^30^.

In the present study, we demonstrated that CST1 affects mitochondrial respiratory chain complex I enzyme activity to elevate OXPHOS levels responsible for the phosphorylation of key proteins in the MEK/ERK pathway. This acts on the mitochondrial respiratory chain to further amplify the effect of CST1 on cancer metastasis, suggesting that CST1 might play a key role in ESCC development by mediating the OXPHOS/MEK/ERK axis.

## Materials and methods

### Cell culture

The human ESCC cell lines TE-1,KYSE150, KYSE520, KYSE140, ECA109 and KYSE410 were purchased from the Chinese Academy of Sciences (Shanghai, China). The cells were cultured in a cell culture incubator at 37 °C with 5% CO_2_.

### Vector transfection

Vector transfection was carried out essentially as described^30^ with slight modifications. The CST1 overexpression/knockdown lentivirus was designed and constructed by the Carrier Biotechnology Company (Fuzhou, China). The vector was VP004-CMV-MCS-3flag-EF1-fLUC-T2A-PURO. The lentivirus was transfected into ESCC cells using Lipofectamine 3000 Reagent (Invitrogen, USA), and the transfection efficiency was detected by RT- qPCR and western blot.

### Real-time PCR

Real-time PCR was carried out essentially as described^30^ with slight modifications. NucleoZOL (Macherey–Nagel, Germany) was used to extract total RNA from ESCC cells, and thereafter 1 μg of extracted total RNA was reversely transcribed into cDNA using the Reverse Transcription PrimeScript™ RT reagent Kit (Takara, Japan). The TB Green Premix Ex Taq Kit (Takara, Japan) was used to detect CST1 mRNA levels. CST1 and GAPDH primers were purchased from Fuzhou Shangya Biotechnology Company, China. The sequences of CST1 primers were CST1-F:5′-GAGGAGACCATGGCCCAGTATC-3′; CST1-R:5′-AGGTCTGCGTTATAGATGCCA-3′.

### Western blot

Western blot was carried out essentially as described^30^ with slight modifications. Total protein was extracted using a whole protein extraction kit (Solarbio, Beijing, China), and protein concentrations were measured using a BCA protein assay kit (Solarbio, Beijing, China). Next, total proteins were separated by SDS/PAGE electrophoresis and then transferred to a PVDF membrane (EpiZyme, Shanghai, China) at 25 °C for 1 h. Overnight incubation with the primary antibody at 4 °C was performed, and then goat anti-rabbit IgG (Beyotime, Shanghai, China) was added at 25 °C for 1 h. Finally, the proteins were detected using a developing solution (Thermo Fisher Scientific, USA). The following primary antibodies were used: CST1 (1:1000; ab307416, Abcam, British), MEK1/2 (1:1000; #9122, CST, USA), p-MEK1/2 (Ser217/221) (1:1000; #9121, CST, USA), p44/42 MAPK (Erk1/2) (1:1000; #9102, CST, USA), p-p44/42 MAPK (Erk1/2) (1:1000; #9101, CST, USA), GRIM19 (1:1000; A18071, Abclonal, China), SDHA (1:1000; A16204, Abclonal, China), UQCRC2 (1:1000; A4184, Abclonal, China), COX IV (1:1000; A6564, Abclonal, China), ATP5A1 (1:1000; A11217, Abclonal, China), VDAC1 (1:1000; A19707, Abclonal, China), MMP2 (1:1000; #4022, CST, USA), and β-actin (1:1000; AF5003, Beyotime, Shanghai, China). All experiments were repeated three times.

### Transwell experiments

Transwell experiments were carried out essentially as described^30^ with slight modifications. No Matrigel matrix (Corning, USA)/matrigel was added to the transwell chambers (Corning, USA) so as to determine cell migration/invasion ability. In 24-well culture plates, 100 μL of medium with a low concentration of fetal bovine serum (FBS) and 8 × 10^4^ cells were added to the upper chamber of the transwell. and 700 μL of medium with a high concentration of FBS was added to the lower chamber and left for 24–48 h. The number of cells passing through the transwell plate was randomly observed after 4% paraformaldehyde fixation and crystalline violet staining. All experiments were repeated three times.

### RNA sequencing

RNA sequencing was carried out essentially as described^17^ with slight modifications. CST1 over-expression cells and negative controls were assembled, and total RNA was prepared using the NucleoZOL reagent. Each sample was prepared in triplicate. RNA-seq was performed using the Illumina platform (Shanghai Genechem Co., China). Differential expression analysis of two groups was then analyzed using the “DESeq2(1.16.1)” and “ClusterProfiler(3.4.4)” software. (https://bioconductor.org/packages/release/bioc/html/DESeq2.html; https://bioconductor.org/packages/release/bioc/html/clusterProfiler.html). Gene Ontology (GO) and Kyoto Encyclopedia of Genes and Genomes (KEGG) enrichment analyses of the DEGs were performed using the Sangerbox platform. Permission has been obtained from Kanehisa laboratories for using KEGG^31^ (www.kegg.jp/kegg/kegg1.html). Values with a fold change of 2 and an adjusted *P*-value of 0.01 were used as the cut-off values.

### Real-time ATP rate detection

Real-time ATP rate detection was performed using an Agilent Seahorse XFe24 instrument (Agilent Technologies Ltd, USA). The XF technique measures the oxygen consumption rate to reflect cellular mitochondrial function and the extracellular acidification rate to reflect cellular glycolytic function. First, the wells of the XF24 cell culture plate were inoculated with a cell suspension at a concentration of 70,000/100 μL, after which each well was refilled with 150 μL of culture medium and incubated overnight in a warm oven. Thereafter, the Seahorse XF assay solution (97 mL RPMI Medium with 1 mL glucose, 1 mL pyruvate, 1 mL glutamine) was prepared and placed in a CO_2_-free incubator at 37 °C. The following day, the assay solution was added to the culture wells. Then, the prepared drugs Oligomycin (1.5 μmol/L) and Rot/AA (0.5 μmol/L) were added to the corresponding dosing wells of the probe plate (56 μL for Oligomycin to well A and 62 μL for Rot/AA to well B). Finally, the culture and probe plates were placed in the instrument, and the measurement were taken at end of the energy metabolism measurement. All experiments were repeated three times.

### Immunofluorescence

First, the crawled slides were fixed with 4% paraformaldehyde in culture plates and then treated with 0.1% Triton X-100 for 15 min; next, the crawled slides were closed with 10% goat serum and incubated with diluted CST1 rabbit primary antibody (1:100; Abcam, UK) and mitochondrial mouse primary antibody (1:200; Abcam, UK) at 4 °C overnight. The following day, the crawls were washed three times with PBS and incubated with secondary antibodies (red fluorescently labeled rabbit secondary antibody and green fluorescently labeled mouse secondary antibody both diluted 1:500, Abcam, UK) at room temperature for 1 h. Finally, the crawls were sealed with a DAPI-containing blocker and observed under fluorescence. All experiments were repeated three times.

### Co-Immunoprecipitation

The overexpression plasmid Flag-CST1 was transfected into ESCC cells. Whole-cell lysates were incubated overnight at 4 °C with anti-Flag (1:50; CST, USA) and anti-CST1 (1:50; Abcam, UK) antibodies or IgG (1:500; Abcam, UK). The complex immune solution was then incubated with protein A/G magnetic beads for 1 h at 25 °C. The cells were then washed to remove unbound immune complexes. For western blot analysis, a metal bath washed off bound immune complexes at 100 °C for 10 min (Pierce Classic Magnetic IP/Co-IP Kit, USA). All experiments were repeated three times.

### Subcellular structure mitochondrial extraction

Five times the volume of pre-chilled mitochondrial extract was added to the collected cells, blown, mixed, and then incubated on ice for 10 min. The supernatant (cytoplasmic component) and precipitate (mitochondria) were collected after centrifugation. Thereafter, the mitochondrial lysate was added to the precipitate and incubated at 4 °C for 20 min. Finally, the lysate was centrifuged with 10,000*g* for 10 min at 4 °C. The supernatant was used as the mitochondrial whole protein extract (subcellular structure mitochondrial extraction kit, Boster, Wuhan, China). All experiments were repeated three times.

### Mitochondria fractionation

Mitochondria fractionation was carried out essentially as described^16^ with slight modifications. Purified mitochondria were suspended in swelling buffer (10 mM KH_2_PO_4_, pH 7.4, containing protease and phosphatase inhibitors) and incubated on ice for 20 min with gentle mixing. Next, the prior mitochondria was mixed with an equal volume of shrinking buffer (10 mM KH_2_PO_4_, pH 7.4, 32% sucrose, 30% glycerol, 10 mM MgCl_2_, and protease and phosphatase inhibitors) on ice for 20 min. Third, the suspension was centrifuged at 10,000*g* for 10 min to obtain the supernatant with the outer membrane and intermembrane-space fractions (OM and IMS) and the pellets with the inner membrane and matrix fractions (IM and MA). We further fractionated the OM&IMS fractions by centrifugation at 150,000*g* for 1 h at 4 °C, and then the supernatant and pellets were collected as IMS and OM, respectively. The supernatant was finally concentrated using a Microcon 10 K centrifugal filter (Millipore, Billerica, MA, USA). All experiments were repeated three times.

### Complex enzyme activity

A mitochondrial respiratory chain complex I activity assay kit (BC0515, Solarbio, Beijing, China) was used to measure mitochondrial OXPHOS complex I enzyme activity. Complex II, III, IV and V activities were measured by assay kits (BC3230, BC3240, BC940, BC1440, Solarbio, Beijing, China), respectively. Absorbance values were determined using a SpectraMax iD5 multifunctional microplate reader (Molecular Devices, USA). All experiments were repeated three times.

### Tumor formation assay for nude mice with ESCC xenograft in situ

Male BALB/c nude mice (4 weeks old) were obtained from Spelford (Beijing) Biotechnology Co. KYSE410 cells transfected with CST1 overexpression/negative control (NC) lentivirus were collected, resuspended in a cell suspension of 8 × 10^7^ cells/mL, and injected into the outer muscle layer of the distal esophagus in the upper part of the gastric cardia. After in situ inoculation (approximately 4 weeks), nude mice with ESCC xenografts in situ were divided into lenti-NC, lenti-CST1, lenti-CST1 + rotenone (1 mg/kg), and lenti-CST1 + PD98059 (1 mg/kg) groups, with four nude mice in each group. The nude mice were subsequently administered drugs, and the metastases of the xenografts were observed by intraperitoneal injection of a luciferase substrate (D-luciferin potassium salt, Beyotime, Shanghai, China) on an IVIS Lumina X5 Small Animal Imaging Analyzer (Perkin Elmer, USA). All experiments were performed in accordance with the Guide for the Care and Use of Animals for Research and approved by the Animal Ethics Committee of Fujian Provincial Hospital (File No. 020-09-014). The study was reported in accordance with ARRIVE guidelines (https://arriveguidelines.org).

### Statistical analysis

Statistical analysis was carried out essentially as described^30^ with slight modifications.All experiments were independently repeated three times. GraphPad Prism V8 software was used for the statistical analysis and charting of experimental data. Data are described as mean ± standard deviation (x ± s). A t-test was used to compare the differences between two groups, and Fisher’s precision test was used to compare the positivity rates. Differences were considered statistically significant at *P* < 0.05.

### Ethics approval and consent to participate

All animal experiments were approved by the Animal Ethics Committee of Fujian Provincial Hospital (approval no. 020–09-014). The study was reported in accordance with ARRIVE guidelines (https://arriveguidelines.org).

## Results

### CST1 regulates OXPHOS in ESCC cells

**Figure 1:**
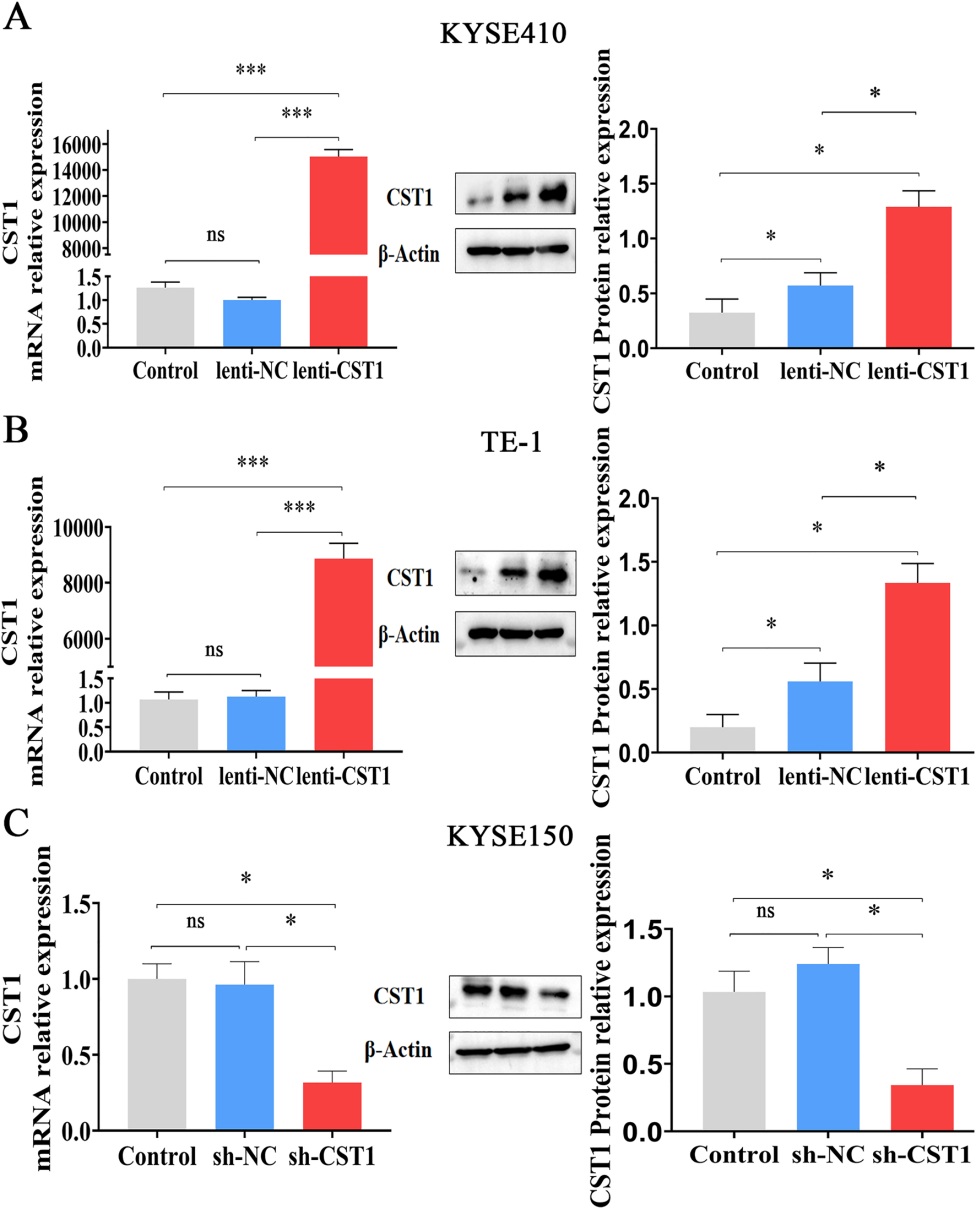
Validation of CST1 overexpression/knockdown effect in ESCC cells. (**A**) CST1 overexpression cell line KYSE410. (**B**) CST1 overexpression cell line TE-1. (**C**) CST1 knockdown cell line KYSE150. (* *P* < 0.01,*** *P* < 0.001,ns means no significance).

**Figure 2:**
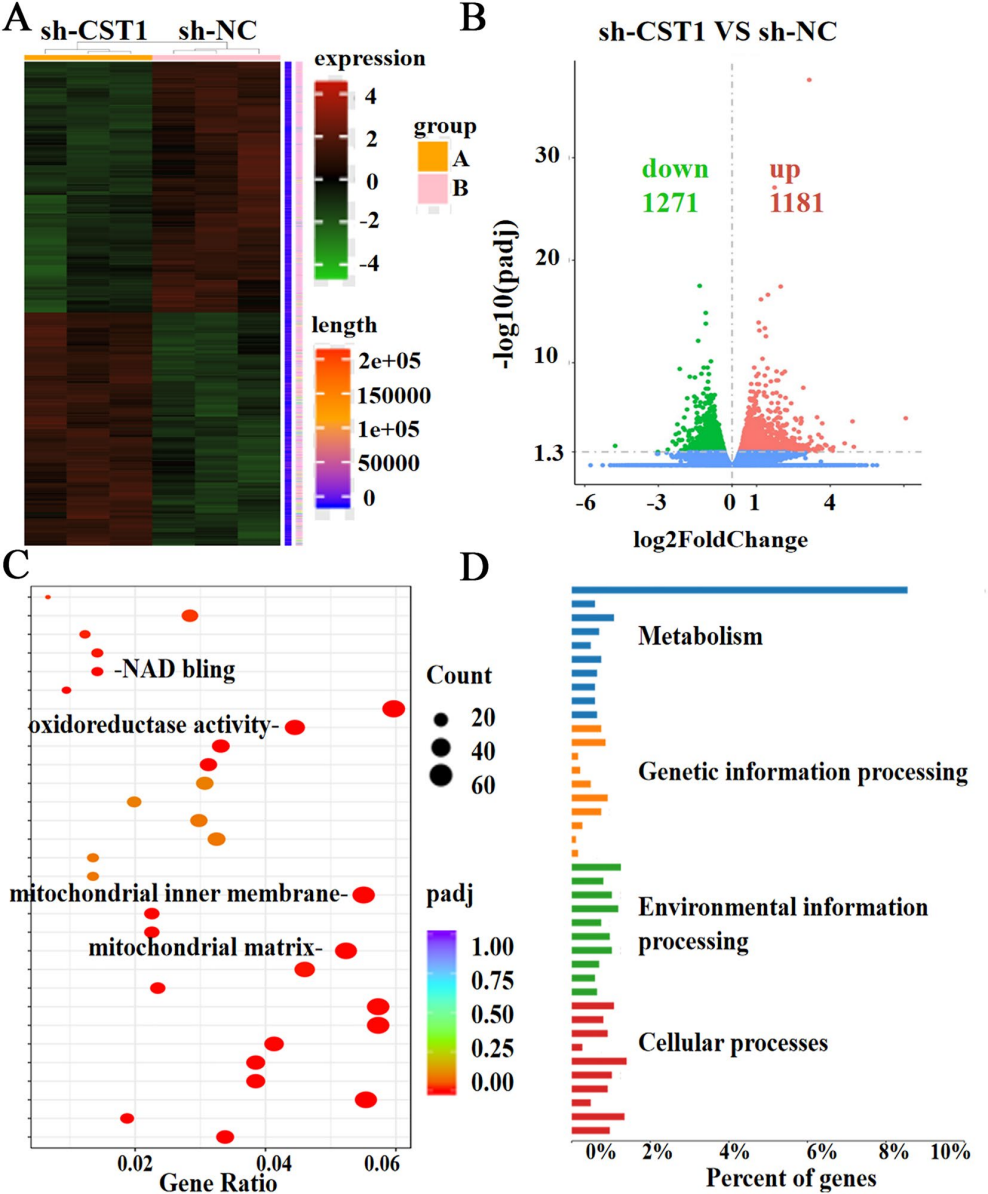
Differential gene analysis. (**A**) Heat map of differential gene expression clustering (**B**) Volcano map of gene differences. (**C**) GO functional enrichment analysis scatter plot. (**D**) KEGG pathway enrichment histogram.

Considering the effect of CST1 on the migration and invasion of ESCC cells observed by constructing CST1 overexpression/knockdown cell lines (Fig. 1A–C) in our pilot study^30^, we conducted a comprehensive survey of the potential mechanism of CST1 in ESCC development by performing transcriptome sequencing of the ESCC cell line KYSE150 with CST1 knockdown using the DESep2 software (Fig. 1C). As a result, a total of 2452 differential genes were obtained, including 1181 genes increased and 1271 genes decreased (Fig. 2A,B); Gene ontology (GO) function and Kyoto Encyclopedia of Genes and Genomes (KEGG) pathway enrichment analyses showed that the differential genes were shown to be closely related to the regulation of cellular metabolic processes and mitochondrial respiratory chain-related oxidoreductase activity, and were significantly enriched in metabolic and tumor-related signaling pathways (Fig. 2C,D). This suggested that CST1 might be involved in regulating mitochondrial OXPHOS in ESCC cells.

### CST1 promotes cellular OXPHOS and mitochondrial complex I enzyme activity in ESCC cells

**Figure 3:**
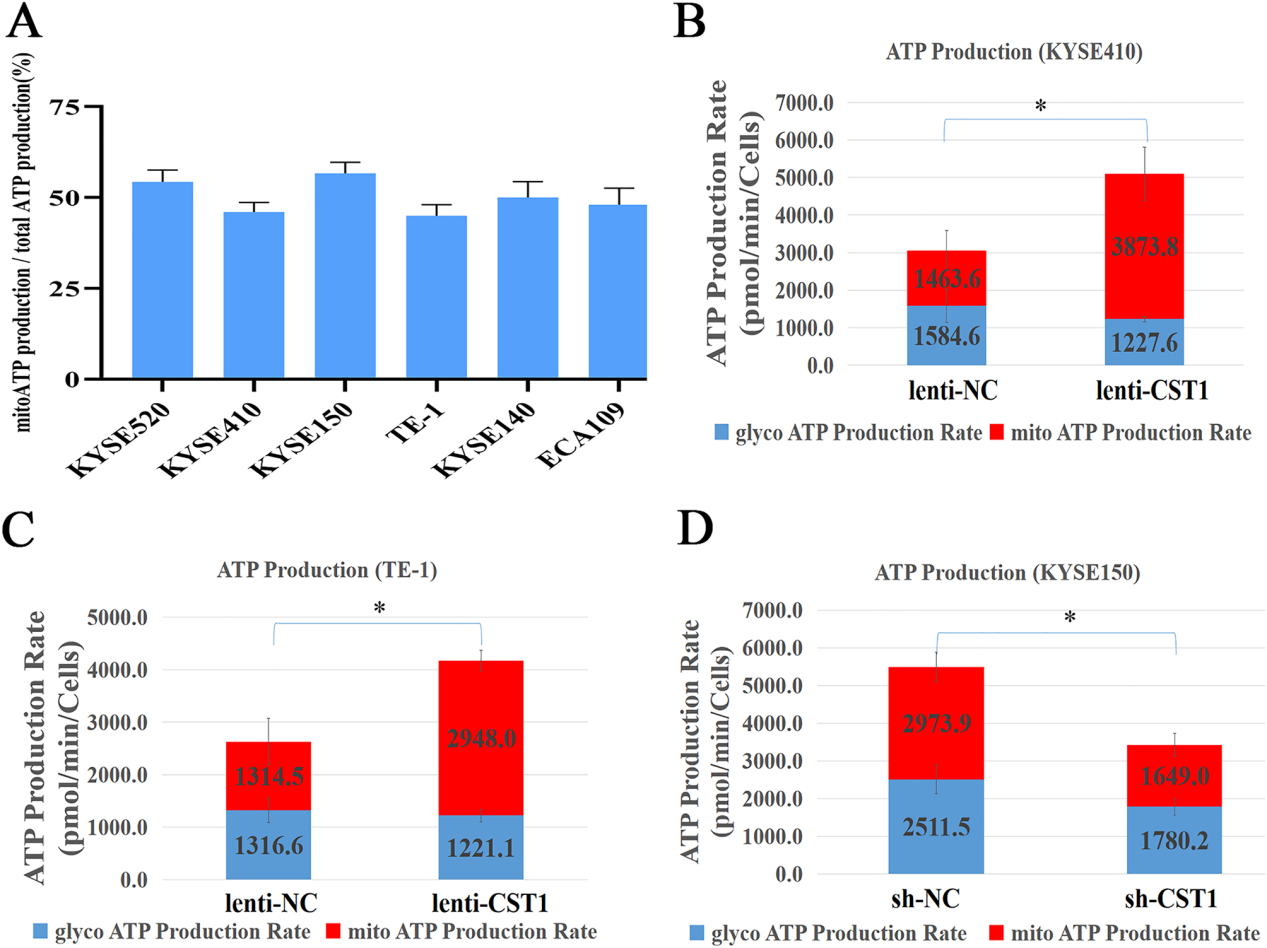
Effects of CST1 on OXPHOS in ESCC cells. (**A**) Ratios of mitochondrial ATP production/total ATP in six common ESCC cell lines. (**B**) ATP production rates of CST1 overexpression cell line KYSE410. (**C**) ATP production rates of CST1 overexpression cell line TE-1. (**D**) ATP production rates of CST1 knockdown cell line KYSE150. (* *P* < 0.01).

**Figure 4:**
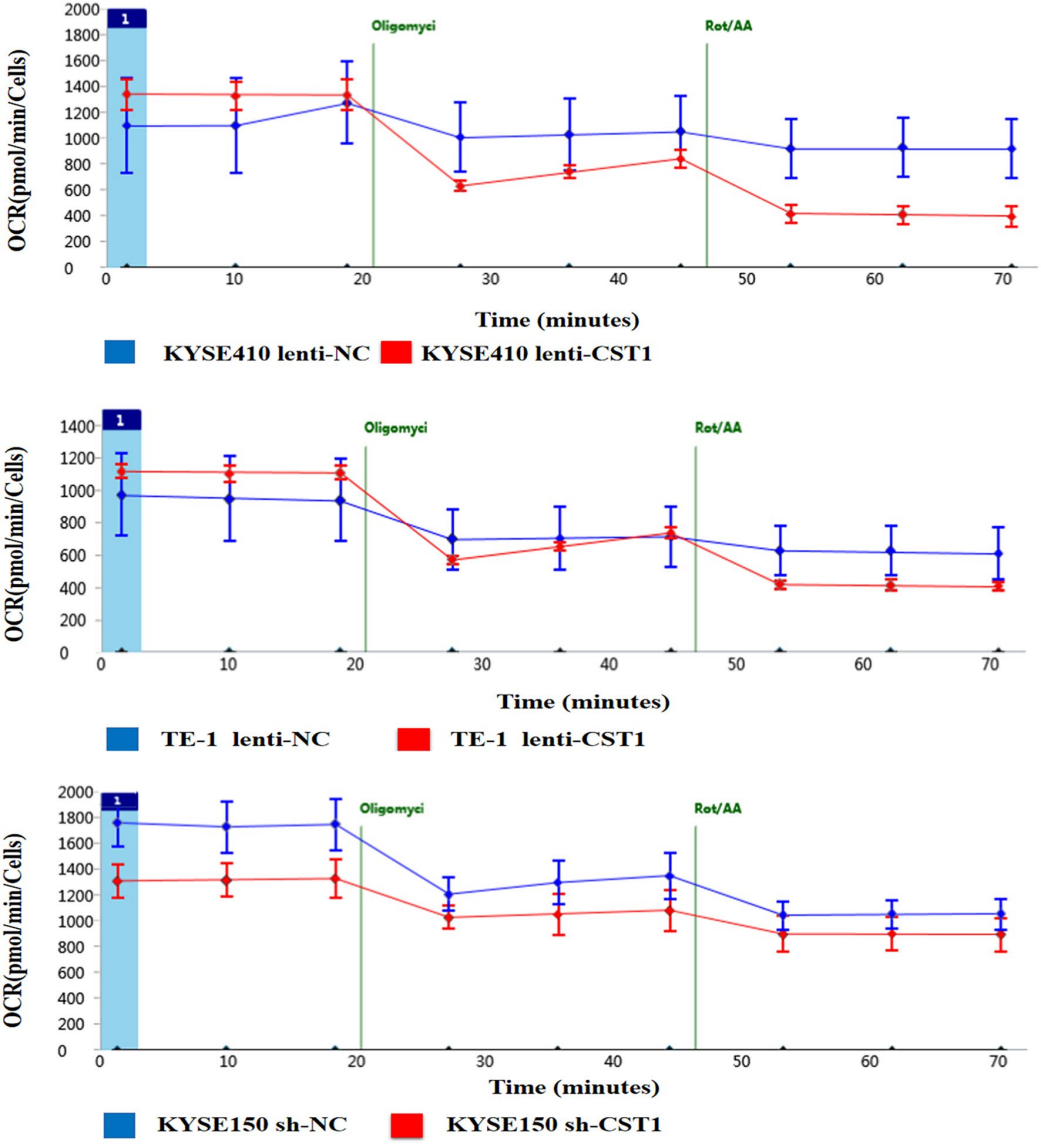
Real-time ATP rates of ESCC cells with CST1 overexpression/knockdown. OCR of cells at baseline and in response to oligomycin and rotenone/antimycin A. (Oligo, an ATP synthase inhibitor; Rotenone, a complex I inhibitor; and Antimycin A, a complex III inhibitor).

To verify whether CST1 affects ESCC cell metabolism, we first defined the energy metabolism of six common ESCC cell lines with mitochondrial ATP/total ATP ratios ranging from 47.4 to 56.5% and mitoATP/glycoATP ratios ranging from 0.9:1 to 1.3:1 (Fig. 3A), suggesting that the main energy supply of ESCC cells is not derived from glycolysis. Moreover, it was shown that the ratios of mitoATP/glycoATP and mitoATP/total ATP in ESCC cell lines KYSE410 and TE-1 with CST1 overexpression were 3.1:1 and 2.4:1, and 75.6% and 70.6%, respectively, both of which were significantly higher than 0.9:1 and 1:1, and 47.4% and 50.0% in the NC group. The opposite change was observed in CST1 knockdown ESCC cell KYSE150, exhibiting a mitoATP/glycoATP ratio of 0.9:1, significantly lower than 1.3:1 in the NC group as well as a significant decrease of ATP level as compared with that in the NC group (Fig. 3B–D). Representative time-dependent changes of the OCR in different groups of cells were shown in Fig. 4 (Observation of cellular responses to different inhibitors of the mitochondrial ETC complexs. Oligo, an ATP synthase inhibitor; Rotenone, a complex I inhibitor; and Antimycin A, a complex III inhibitor. Figure 3B–D are a quantification of Fig. 4).

**Figure 5:**
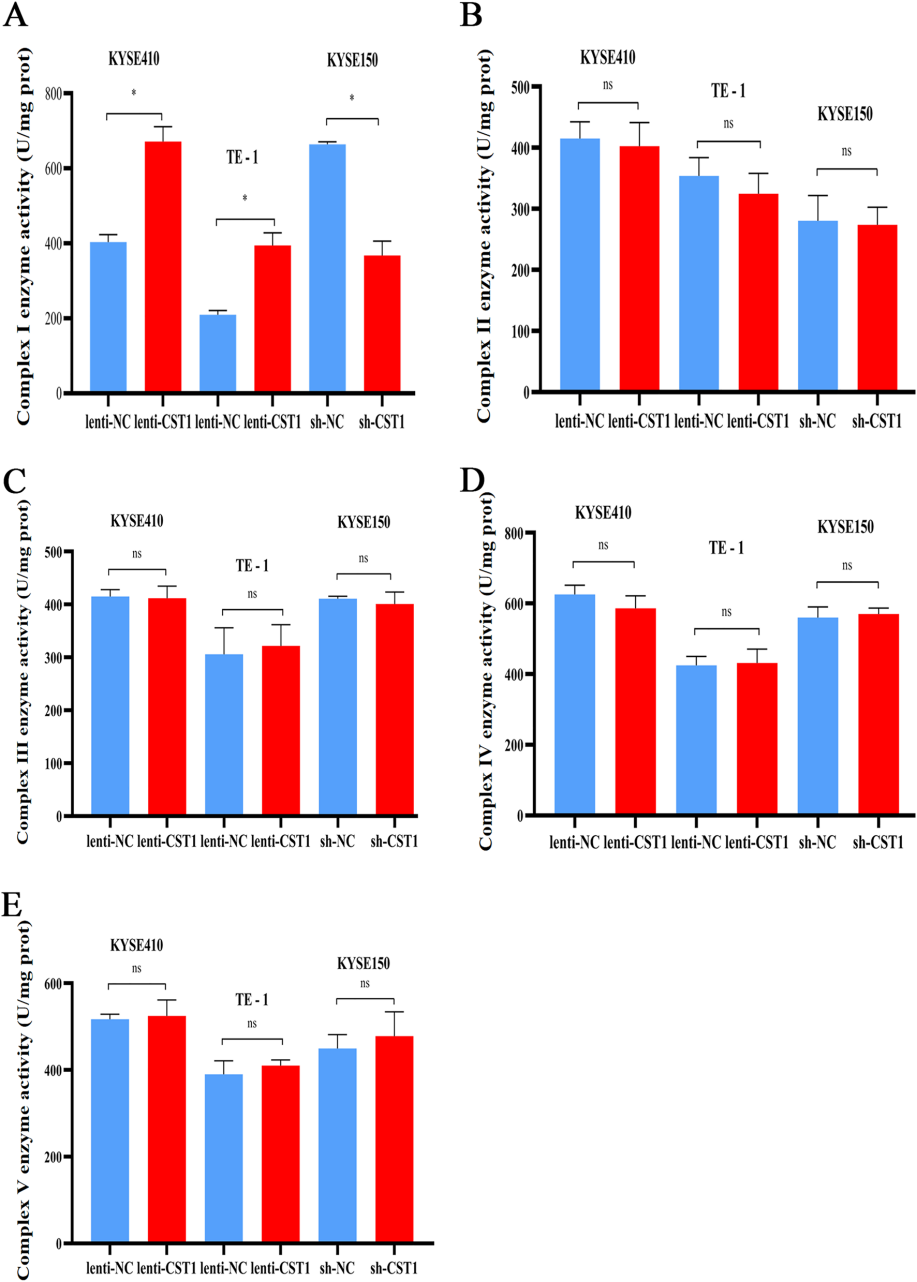
Effect of CST1 on the enzymatic activity of each complex of mitochondrial respiratory chain in ESCC cells. (**A**) Complex I activity. (**B**) Complex II activity. (**C**) Complex III activity. (**D**) Complex IV activity. (**E**) Complex V activity. (** *P* < 0.01, ns means no significance).

It is well known that ATP production by cellular mitochondrial OXPHOS mainly depends on the respiratory chain, which comprises five protein complexes on the mitochondrial membrane. To further investigate the effect of CST1 on the mitochondria, we measured the activity of each complex using an enzymatic assay. The results showed that CST1 expression significantly upregulated the activity of mitochondrial respiratory chain complex I in ESCC cells (Fig. 5A), while not changing the activities of complexes II, III, IV, and V (Fig. 5B–E), suggesting that CST1 expression might elevate the OXPHOS levels by upregulating the activity of mitochondrial respiratory chain complex I in ESCC cells.

### CST1 interacts with GRIM19 to regulate OXPHOS in ESCC

**Figure 6:**
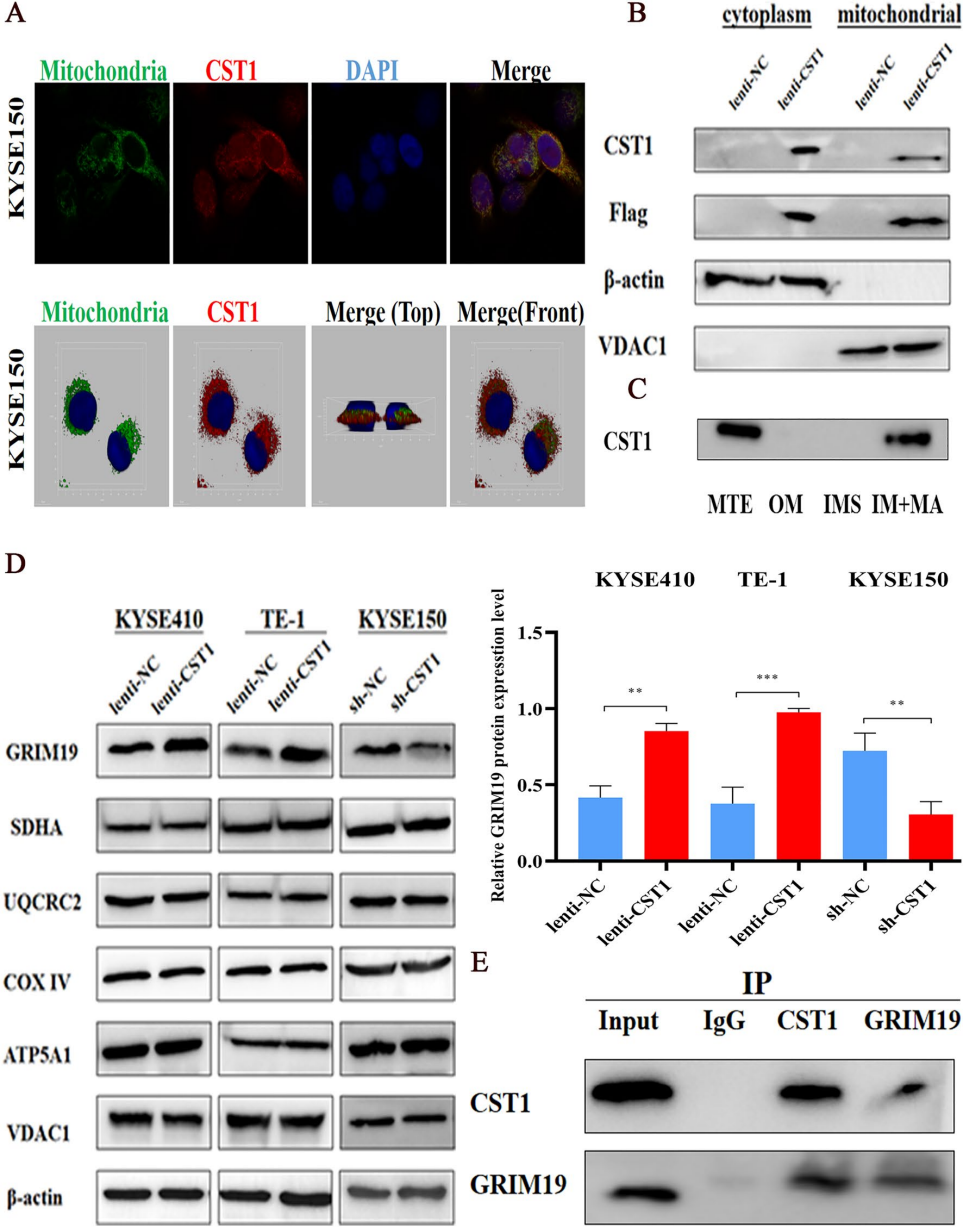
Distribution of CST1 protein in ESCC cells. (**A**) Cytosolic fluorescence staining of co-localization of CST1 with mitochondria in the cytoplasm. (**B**) Western blot detection of cytoplasmic and mitochondrial CST1 expression. (**C**) Western blot detection of CST1 protein expression in mitochondrial outer membrane (OM), membrane gap (IMS), and inner membrane + matrix (IM + MA). (**D**) Changes of subunits of the mitochondrial complex. (**E**) Co-Immunoprecipitation (CO-IP) of interaction of CST1 with GRIM19. (** *P* < 0.01,*** *P* < 0.001).

To further confirm the relationship between CST1 and mitochondrial OXPHOS, we first defined the colocalization of CST1 and mitochondria existed in ESCC cells by cytofluorimetric staining (Fig. 6A). By isolating the different components of the cells, we also found that CST1 protein was present in both mitochondrial extracts (Fig. 6B) and mitochondrial components (inner membrane + matrix) by western blotting (Fig. 6C). Subsequently, we examined the subunits of each complex-related protein in the mitochondrial respiratory chain: GRIMI19 (complex I subunit), SDHA (complex II subunit), UQCRC2 (complex III subunit), COX IV (complex IV subunit), ATP5A1 (complex V subunit), and VDAC1 (involved in mitochondrial and cytoplasmic ATP transport). GRIM19 expression was upregulated in ESCC cells with CST1 overexpression and downregulated in ESCC cells with CST1 knockdown, whereas no significant changes were observed in other respiratory chain complex subunits (Fig. 6D). Finally, to explore the relationship between CST1 and GRIM19 protein, we detected GRIM19 in the protein eluate after pulling against CST1, and CST1 in the protein eluate after pulling against GRIM19 by co-immunoprecipitation (CO-IP), indicating the mutual binding of both proteins (Fig. 6E). These results demonstrated that CST1 might upregulate the activity of mitochondrial respiratory chain complex I by interacting with the subunit GRIM19.

### CST1 promotes cell metastasis by mediating the OXPHOS/ MEK/ERK axis in ESCC

**Figure 7:**
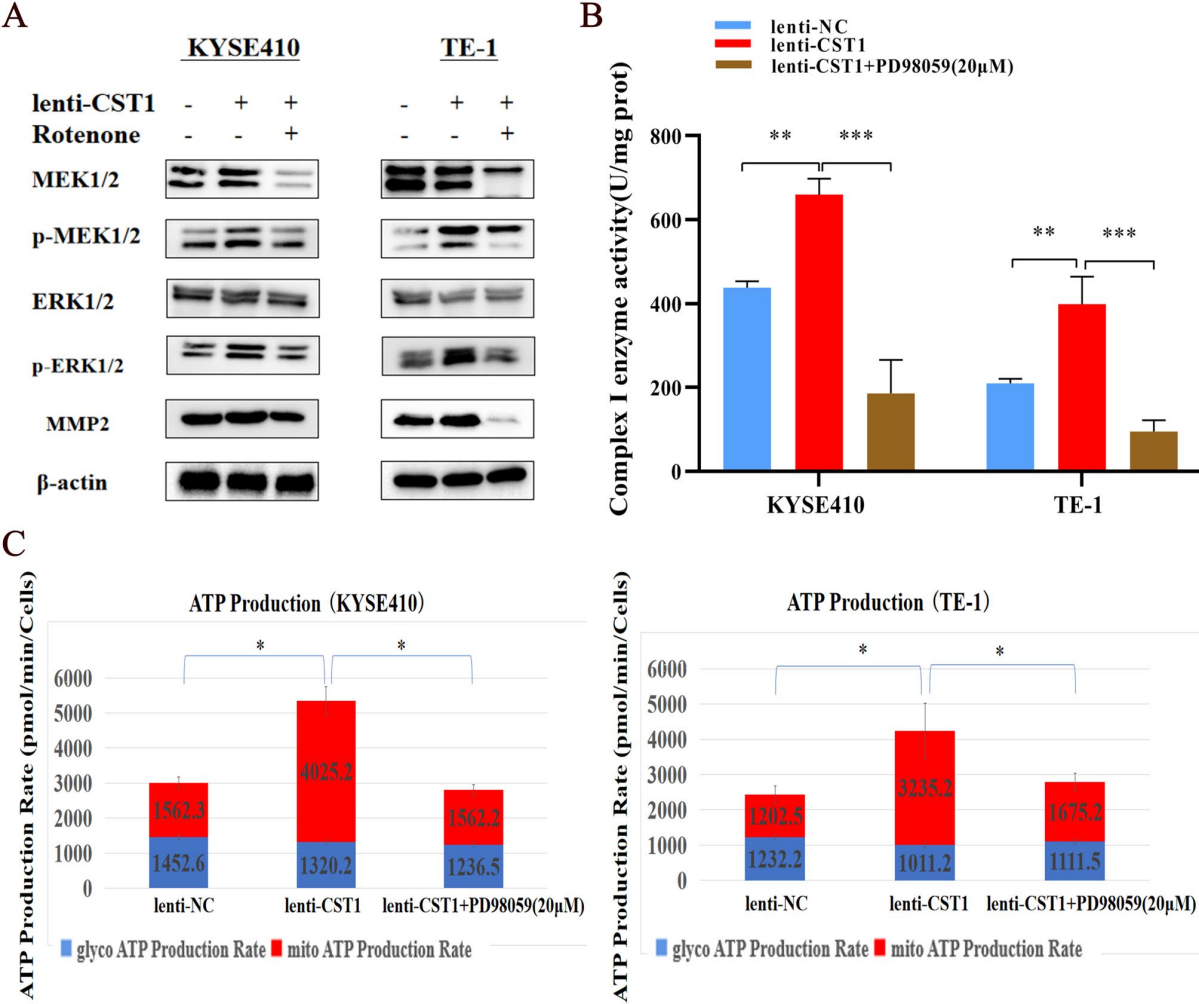
Interaction between OXPHOS and MEK/ERK pathway. (**A**) Changes of MEK/ERK pathway after the addition of Rotenone (2 μM, the complex I enzyme inhibitor). (**B**) Changes of complex I enzyme activity after the addition of PD98059 (20 μM, MEK/ERK pathway inhibitor). (**C**) Changes of cellular ATP production after addition of PD98059 (20 μM, MEK/ERK pathway inhibitor). (* *P* < 0.05,** *P* < 0.01,*** *P* < 0.001).

**Figure 8:**
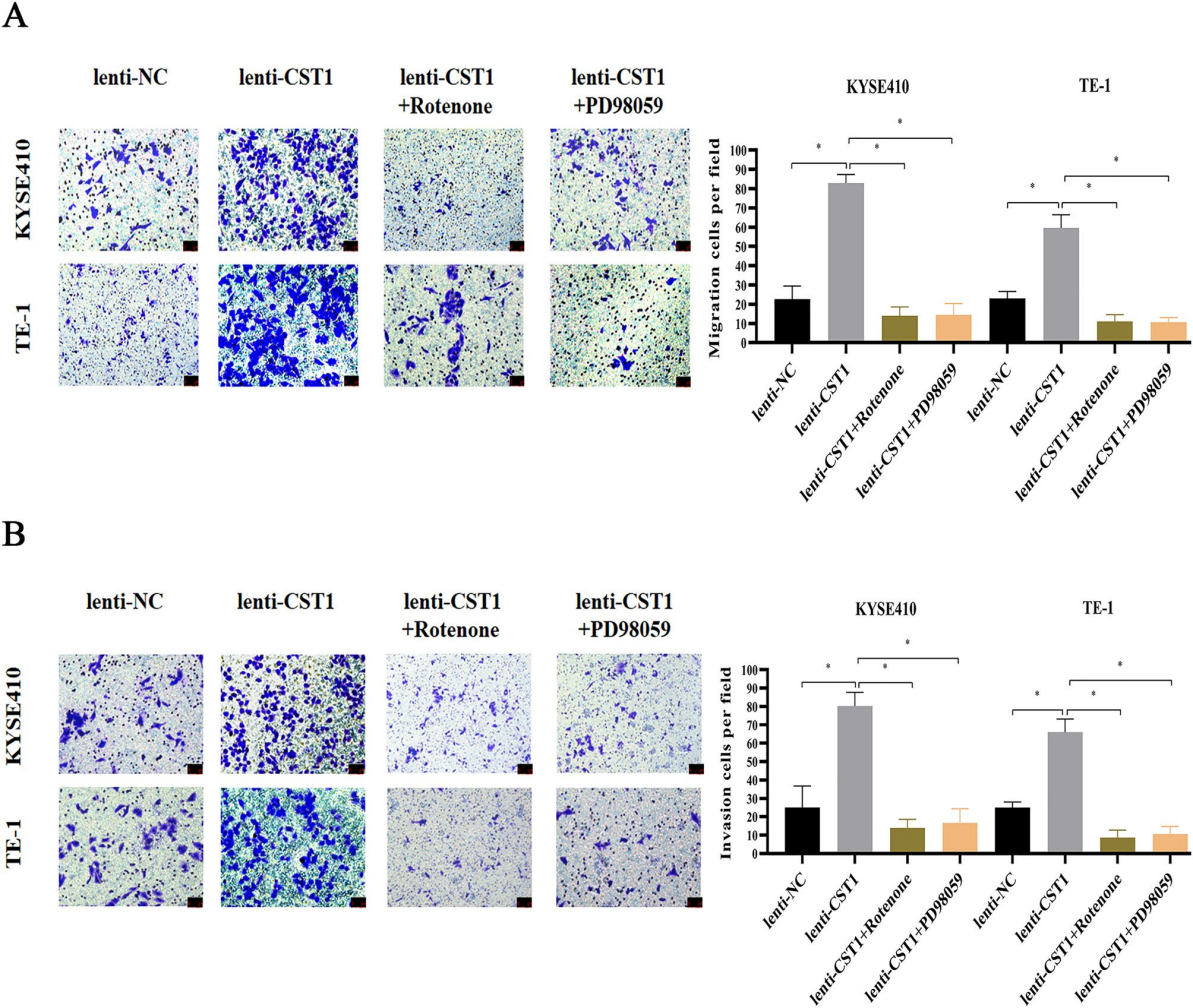
Effects of CST1 on migration and invasion of ESCC cells (× 200). (**A**) Changes of cell migration ability after the additions of inhibitor Rotenone (2 μM) and PD98059 (20 μM) (× 200). (**B**) Changes of cell invasion ability after the additions of inhibitor Rotenone (2 μM) and PD98059 (20 μM) (× 200). (* *P* < 0.05).

Considering the effect of CST1 on promoting the phosphorylation levels of MEK/1/2/ERK1/2 and EMT-related protein expression in our pilot study^30^, we explored the relationship between OXPHOS and the MEK/ERK signaling pathway in ESCC cells, by adding rotenone^17^ (a mitochondrial respiratory chain complex I inhibitor) to the ESCC cell lines KYSE410 and TE-1 with CST1 overexpression. The expression of p-MEK1/2, p-ERK1/2, MMP2 (EMT-related protein) in ESCC cells was obviously downregulated by the inhibition of mitochondrial OXPHOS (Fig. 7A), indicating that CST1 might activate the MEK/ERK pathway by elevating mitochondrial OXPHOS levels in ESCC cells. Surprisingly, cellular OXPHOS was significantly inhibited by the addition of PD98059 (MEK/ERK signaling pathway inhibitor), evidenced by a decrease in mitochondrial complex I enzyme activity (Fig. 7B) and total ATP production level (Fig. 7C), suggesting that there might be a reciprocal regulatory relationship between OXPHOS and the MEK/ERK pathway in ESCC cells. We then defined the effects of the OXPHOS and MEK/ERK pathways on promoting the migration and invasion of ESCC cells with CST1 overexpression, as evidenced by the significant decrease in migration and invasion of cells by the addition of the inhibitors rotenone and PD98059, respectively (Fig. 8A,B). Therefore, we proposed that CST1 might mediate an OXPHOS/MEK/ERK axis to promote the metastasis of ESCC cells based on our in vitro study.

### CST1 promotes ESCC cell metastasis in nude mice

**Figure 9:**
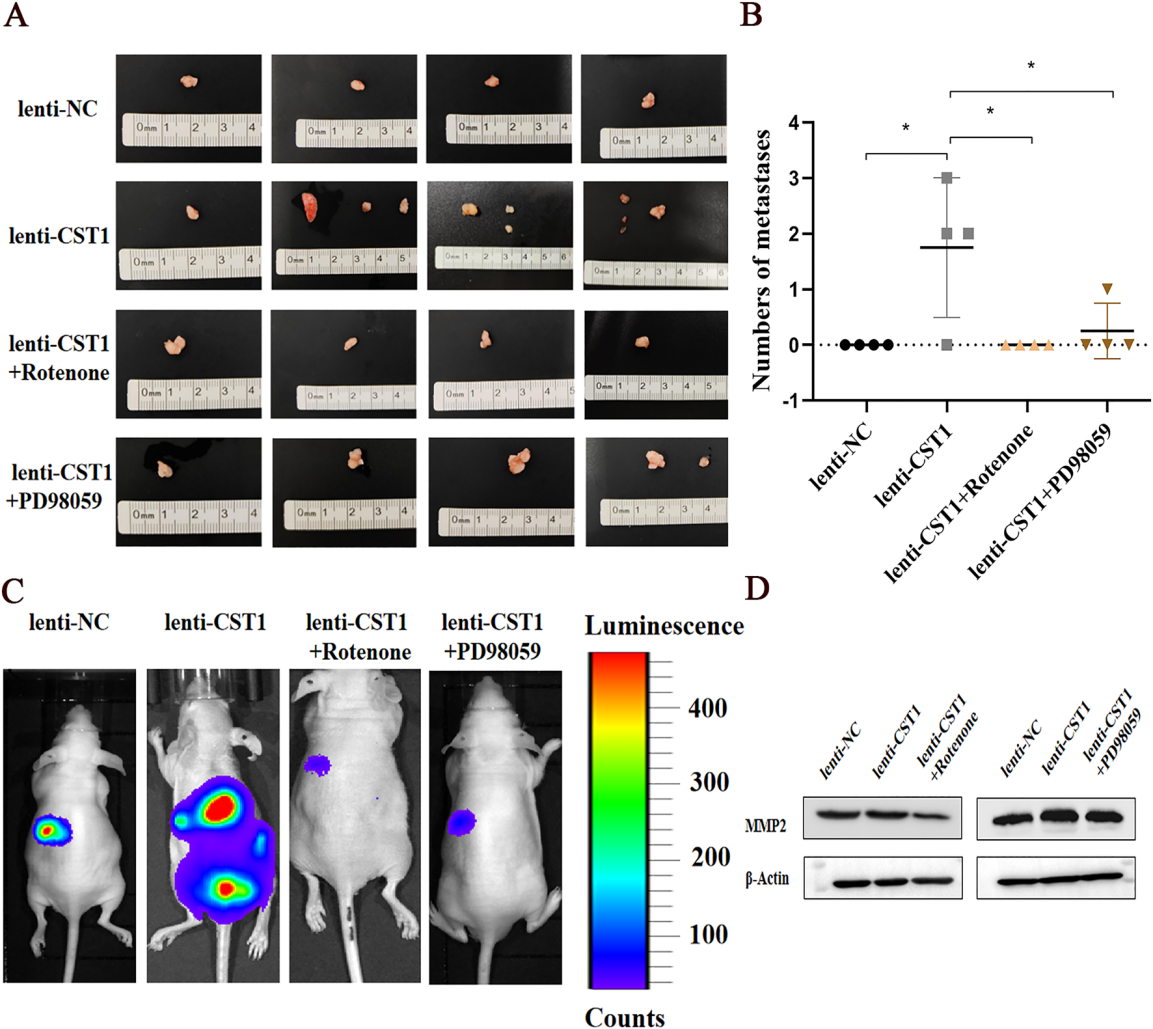
CST1 promoted ESCC cell metastasis in nude mice. (**A**, **B**) Differences of numbers of metastases. (**C**) Distributions of metastases under fluorescence. (**D**) MMP2 protein expression of xenografts. (* *P* < 0.05).

To further verify the effect of CST1 on the metastasis of ESCC cells in vivo, we generated nude mice with ESCC xenografts in situ by injecting the ESCC cell line KYSE410 with CST1 overexpression into the outer muscle layer of the distal esophagus in the upper part of the gastric cardia.Then, nude mice were divided into four groups including the lenti-NC group, lenti-CST1 group, lenti-CST1 + rotenone group, and lenti-CST1 + PD98059 group, with four nude mice in each group. There were significantly more metastases, higher total fluorescence intensity, and MMP2 protein expression in ESCC xenografts in the lenti-CST1 group than in the lenti-NC group, without significant differences in the size, volume and weight of the tumor tissues, elucidating the effect of CST1 on promoting the metastasis of ESCC cells in vivo (Fig. 9A–D). In addition, the number of metastases, total fluorescence intensity, and MMP2 protein expression of ESCC xenografts in the lenti-CST1 + rotenone and lenti-CST1 + PD98059 groups were significantly reduced as compared with those in the lenti-CST1 group (Fig. 9A–D), confirming that CST1 might promote metastasis of ESCC cells via the OXPHOS and MEK/ERK pathways in vivo.

## Discussion

Recent studies have indicated that CST1 is highly ectopically expressed in cancerous tissues and plays a potentially oncogenic role in some malignancies^32,33^. For example, Liu et al^25^ reported that CST1 is involved in the development of ER + breast cancer by regulating the ERα/ PI3K/Akt /ERα loop. Besides, CST1 was found to promote gastric cancer by mediating Wnt/β-catenin/TCF signaling as a downstream effector of transcription factor TCF^34^. CST1 has also been shown to have oncogenic potential in ESCC development by our pilot study, as demonstrated by its aberrantly high expression and effect of CST1 on cell migration and invasion via the MEK/ERK signaling pathway in ESCC^30^. Therefore, we conducted a comprehensive survey of the potential mechanism of CST1 in ESCC development by performing the transcriptome sequencing of ESCC cells with CST1 knockdown, and surprisingly discovered that the differential genes were closely related to the regulation of cell metabolic processes and mitochondrial respiratory chain-related oxidoreductase activity, and were significantly enriched in metabolic and tumor-related signaling pathways, suggesting that CST1 might be involved in mediating mitochondrial OXPHOS in ESCC cells.

Considering that cancer cells require large amounts of ATP to meet their energy demands for survival, reprogramming of cellular energy metabolism occurs during cancer development. For example, many cancer cells rely mainly on aerobic glycolysis for energy and biomass production, known as the Warburg effect^35–37^. These cancer cells exhibit abnormal mitochondrial inactivation with a shift in mitochondria-dependent OXPHOS to aerobic glycolysis as the main energy source, even under aerobic conditions^38^. Nevertheless, increasing evidence indicates that mitochondrial OXPHOS also plays a key role in cancer progression, manifested as some cancer cells relying on mitochondrial OXPHOS to obtain ATP more efficiently than glycolysis, providing a promising approach for cancer treatment in recent years^19,39^. For example, increased mitochondrial OXPHOS levels are responsible for proliferation, metastasis, and resistance to chemotherapeutic agents in some malignancies, whereas the inhibition of mitochondrial respiratory chain complex I significantly reduces the electron transport capacity and aggressiveness of cancer cells^16,18,40^. Thus, it is reasonable to deduce that mitochondrial OXPHOS is crucial for the survival of some cancer cells, with OXPHOS other than glycolysis as the main source of energy.

In particular, by detecting the real-time ATP rate of six common ESCC cell lines, we determined that the main source of energy supply for ESCC cells is derived from OXPHOS rather than glycolysis, as evidenced by mitochondrial ATP/total ATP ratios ranging from 47.4% to 56.5% and mitochondrial ATP/glycolytic ATP ratios ranging from 0.9:1 to 1.3:1. Moreover, markedly higher ratios of mitochondrial ATP/glycolytic ATP as well as higher ATP levels were observed in the ESCC cell lines KYSE410 and TE-1 with CST1 overexpression as compared with those in the NC group. Together with the opposite changes observed in ESCC cells with CST1 knockdown, this elucidated the effect of CST1 expression on enhancing mitochondrial OXPHOS in ESCC cells. Thus, the mechanism by which CST1 enhances mitochondrial OXPHOS in ESCC cells warrants further investigation. Encouragingly, by cytofluorimetric staining and western blot assay, we determined the colocalization of CST1 and mitochondria, and the presence of CST1 protein in the inner membrane and matrix of the mitochondrial component. Furthermore, the CO-IP assay revealed that CST1 was bound to the protein GRIM19, a key mitochondrial respiratory chain complex I subunit, suggesting that CST1 might enhance the activity of mitochondrial respiratory chain complex I by interacting with subunit GRIM19.

**Figure 10:**
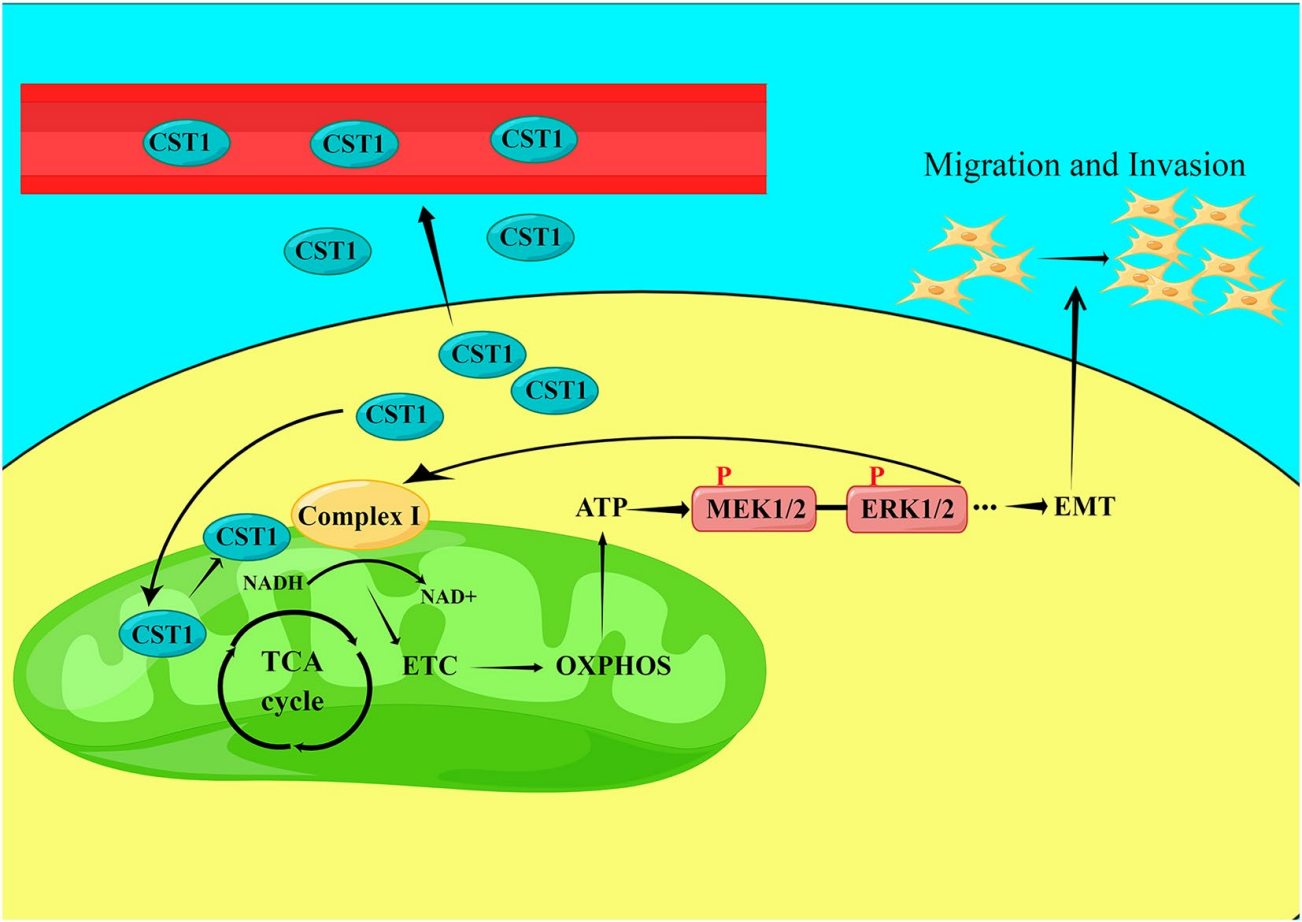
A proposed working model for the mechanism by which CST1 mediates an OXPHOS/MEK/ERK axis to promote ESCC cell migration and invasion (Picture produced by figdraw).

Based on the remarkable effect of CST1 in upregulating the phosphorylation of MEK/1/2/ERK1/2 observed in our pilot study^30^ and the potential of the activated MEK/ERK pathway to enhance the activity of the cellular mitochondrial respiratory chain^41^, we deduced that there may be a crosstalk between mitochondrial OXPHOS and the MEK/ERK pathway in ESCC cells with CST1 overexpression. Encouringly, we achieved the expected results, which showed that p-MEK1/2/p-ERK1/2 expression was significantly reduced after the addition of the complex I inhibitor rotenone. In addition, the mitochondrial respiratory chain complex I activity as well as ATP levels and mitochondrial ATP/glycolysis, were obviously reduced after the addition of the p-MEK1/2/p-ERK1/2 inhibitor PD98059, suggesting that CST1 might have a reciprocal regulatory relationship existed between OXPHOS and the MEK/ERK pathway in ESCC cells. Finally, the detection of nude mice with ESCC xenografts in situ divided into four groups including lenti-NC, lenti-CST1, lenti-CST1 + rotenone and lenti-CST1 + PD98059 groups demonstrated the potential of CST1 in ESCC metastasis by regulating of OXPHOS and MEK/ERK pathways in vivo. In summary, to the best of our knowledge, this study is the first to reveal the oncogenic role of CST1 in ESCC by enhancing mitochondrial respiratory chain complex I enzyme activity to mediate the OXPHOS/MEK/ERK axis responsible for cell migration and invasion (Fig. 10), suggesting that CST1/OXPHOS might be a promising target for ESCC treatment. Nevertheless, we must acknowledge that the mechanism by which CST1 expression leads to the upregulation of mitochondrial respiratory chain complex I activity by interacting with GRIM19 remains unknown and needs to be uncovered in future studies.

### Supplementary Information

Supplementary Information.

## Data Availability

The datasets used and/or analyzed during the current study are available from the corresponding author on reasonable request.
