# Supplementary figures and images for: Cysteine protease inhibitor 1 promotes metastasis by mediating an oxidative phosphorylation/MEK/ERK axis in esophageal squamous carcinoma cancer
Source: Sci Rep. 2024 Feb 29;14:4985. doi: 10.1038/s41598-024-55544-1 (PMC10904862; doi:10.1038/s41598-024-55544-1)

Fig 1A

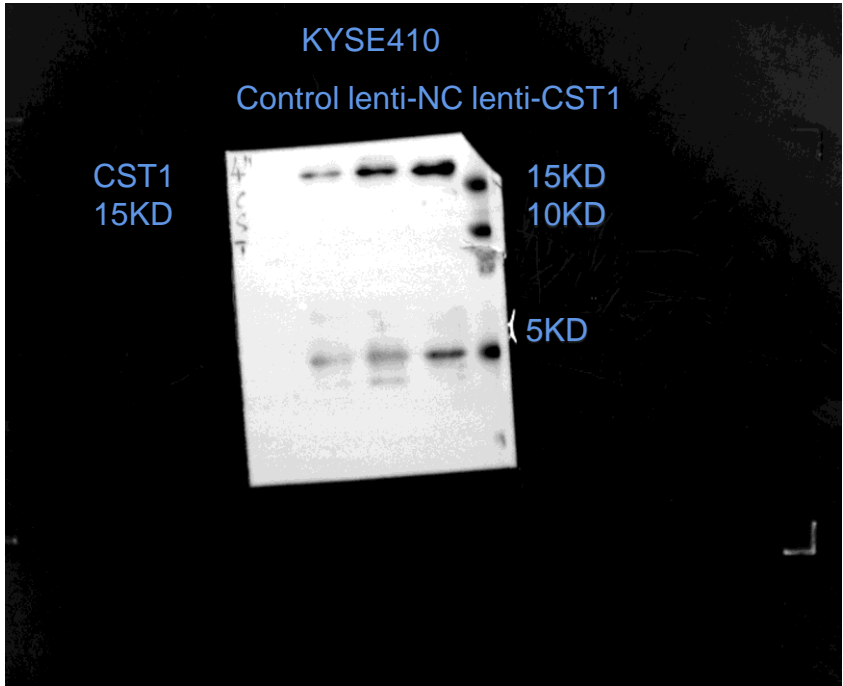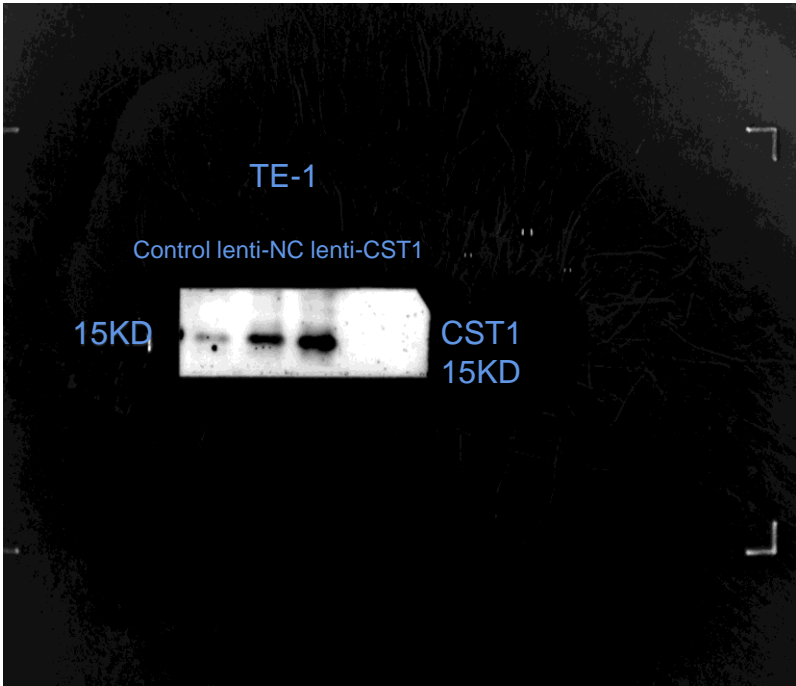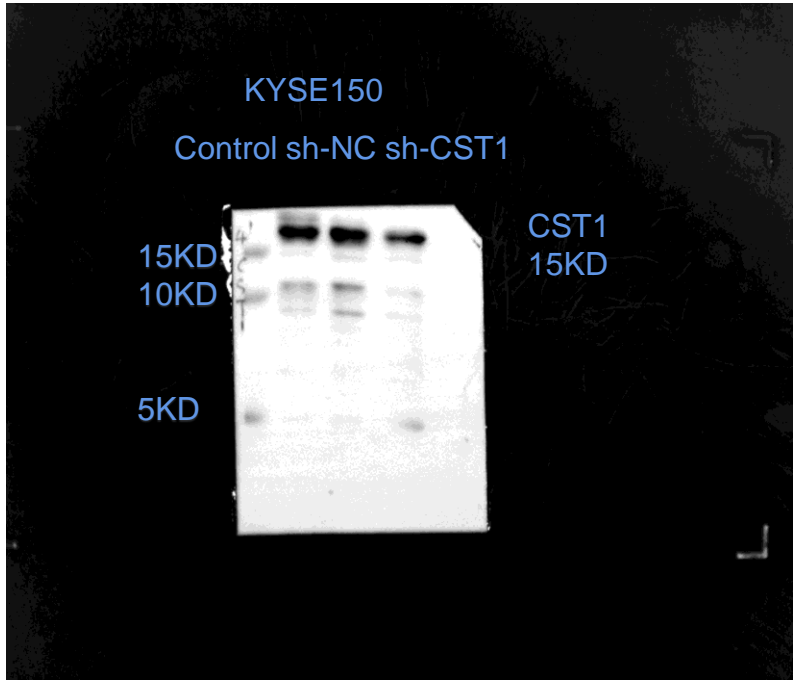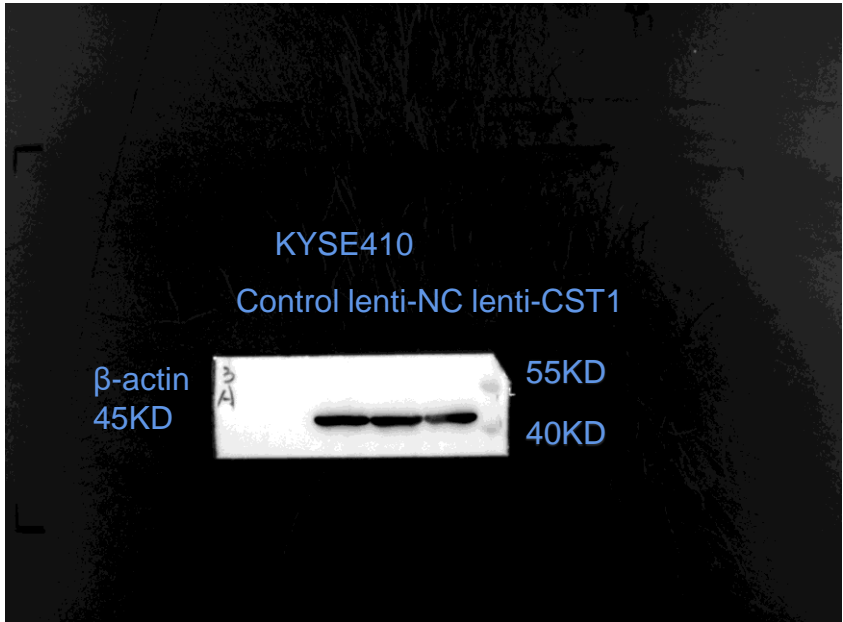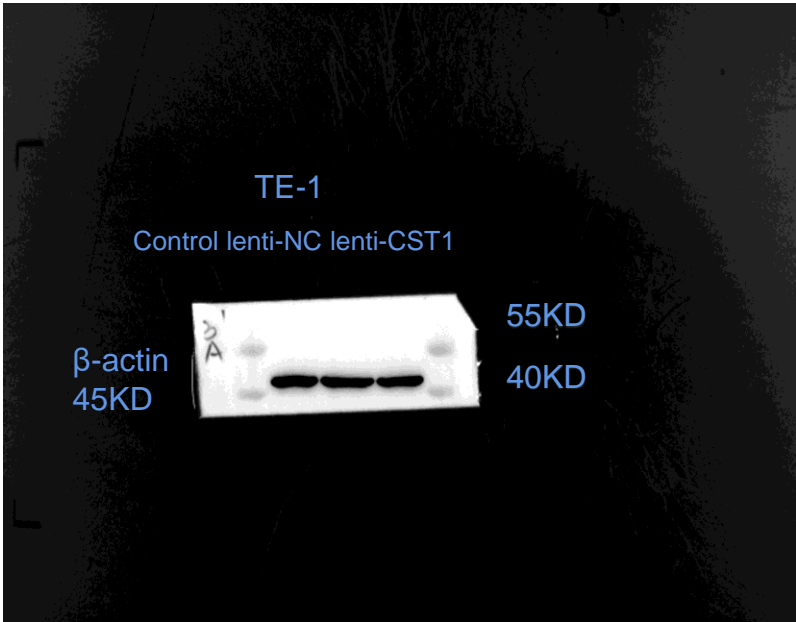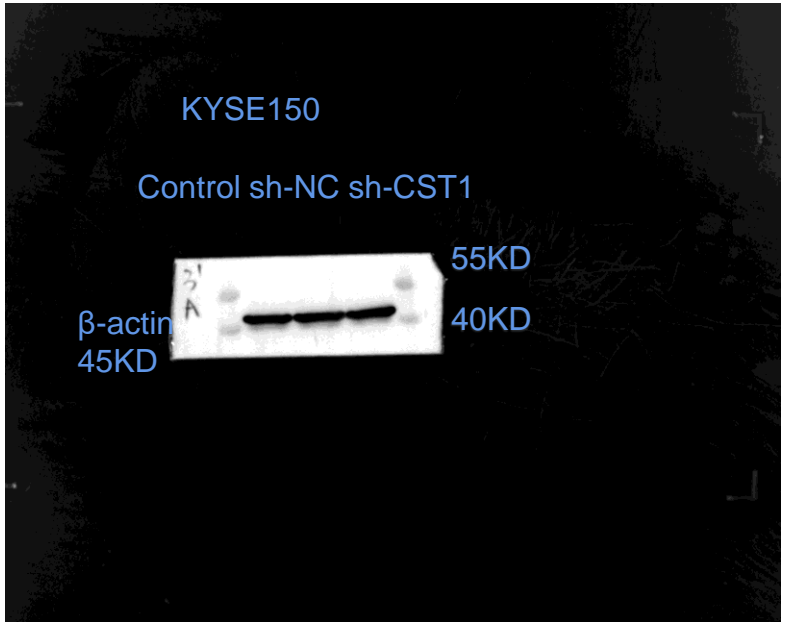

Fig 6B

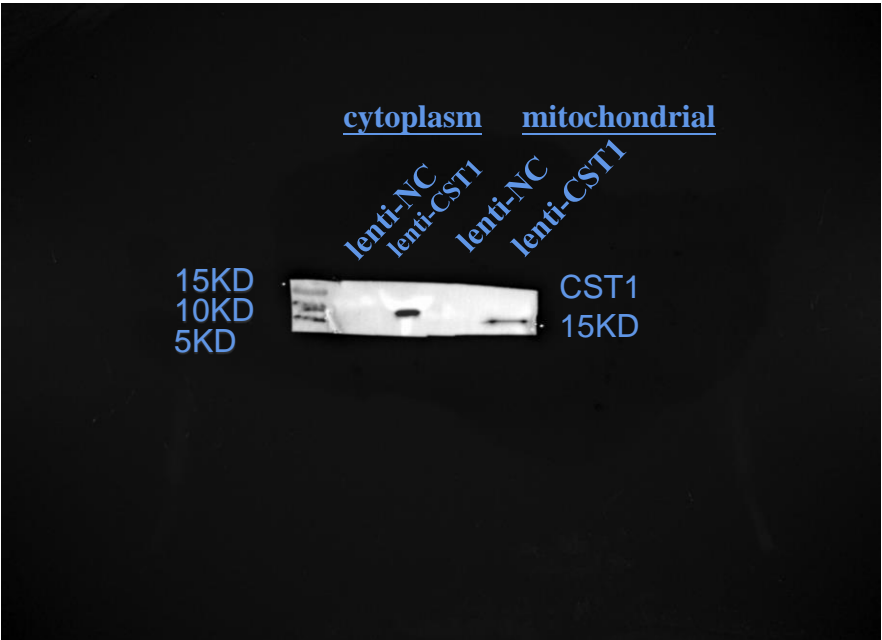

Fig 6C

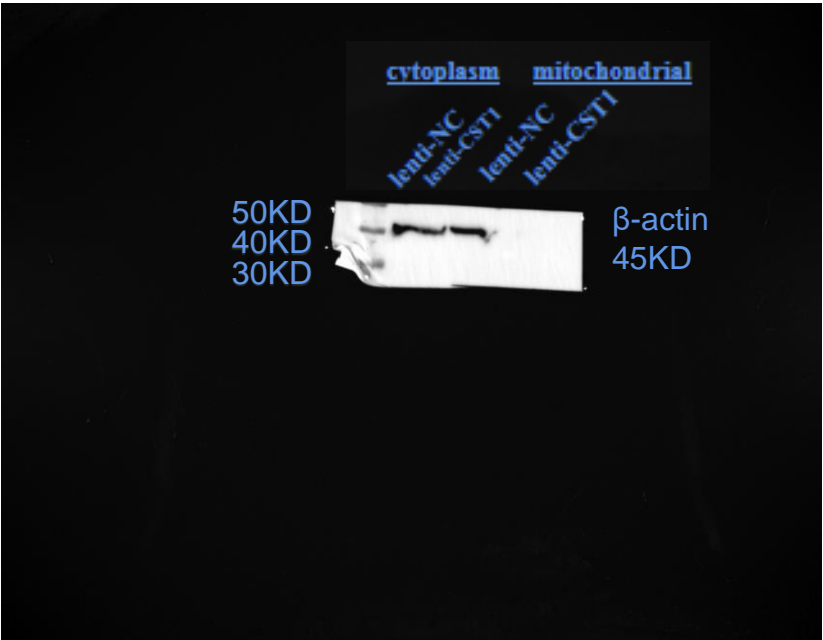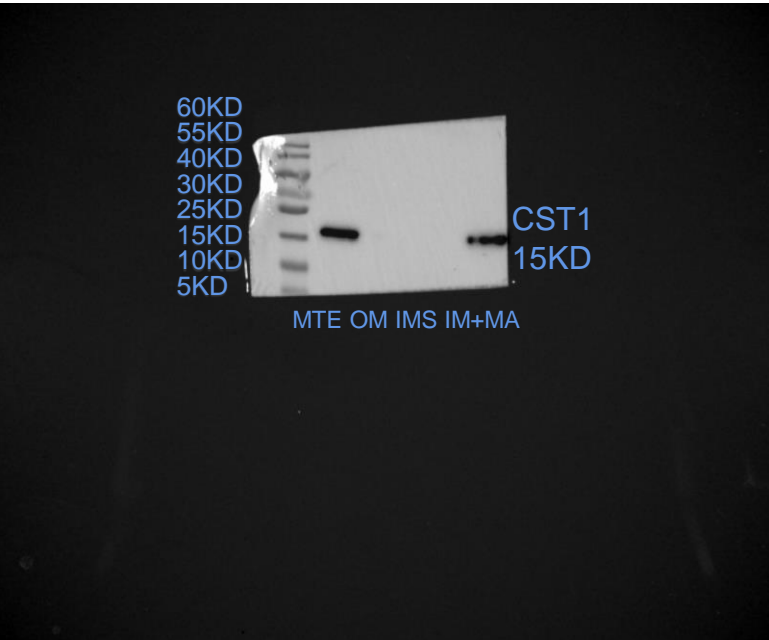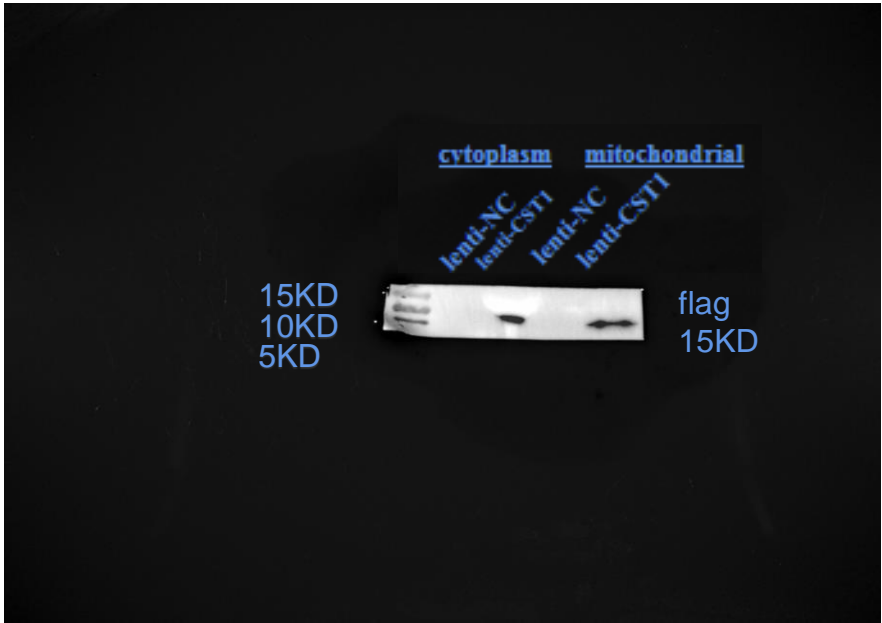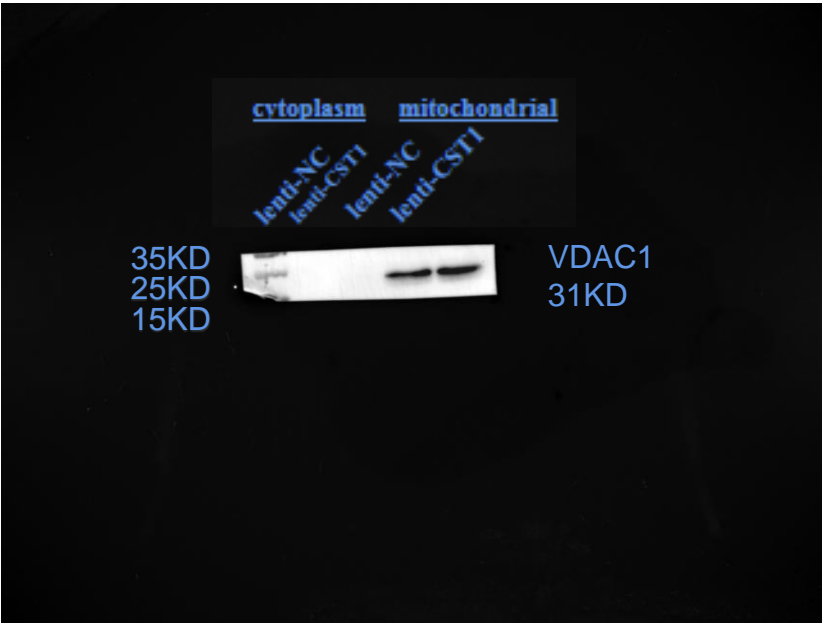

Fig 6D

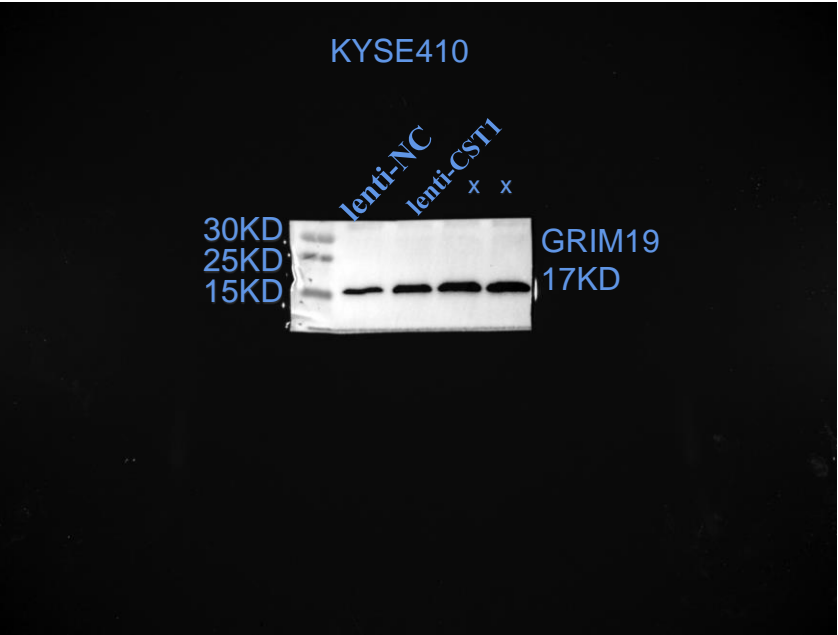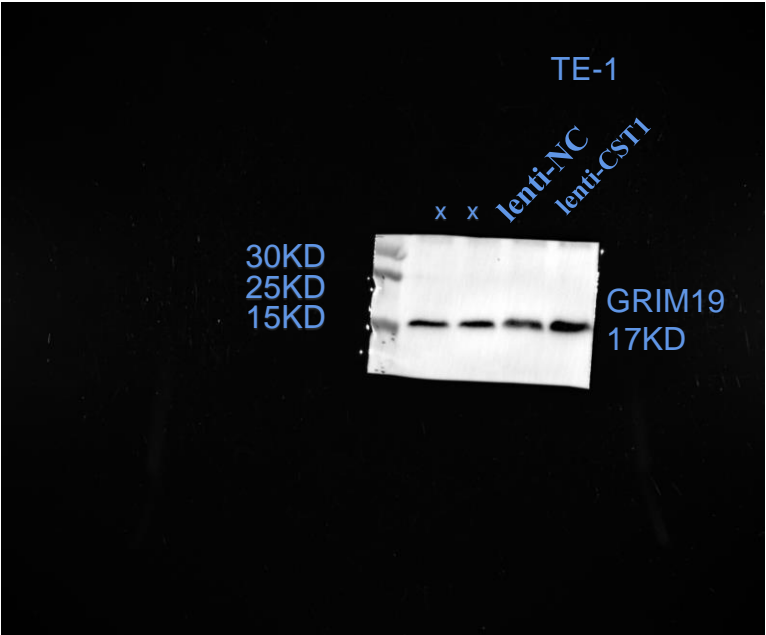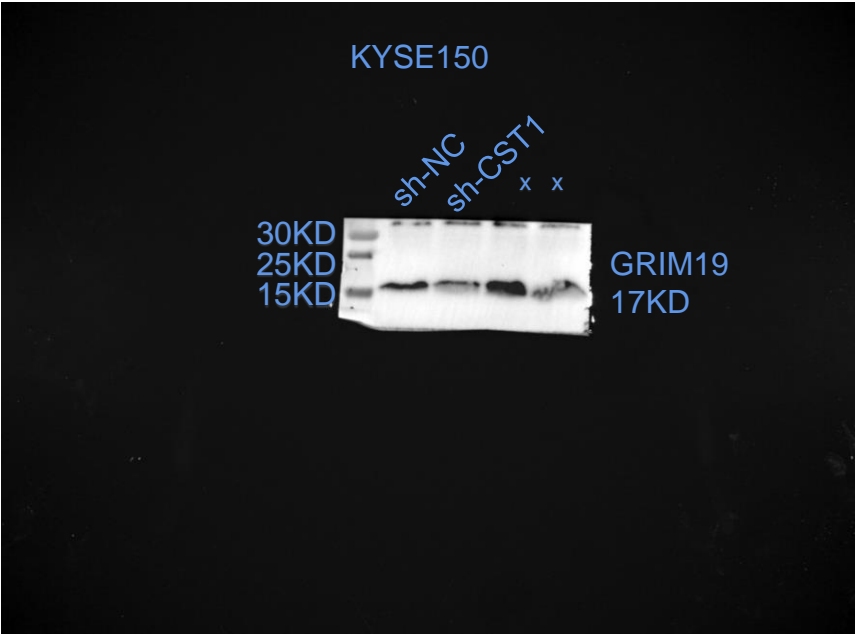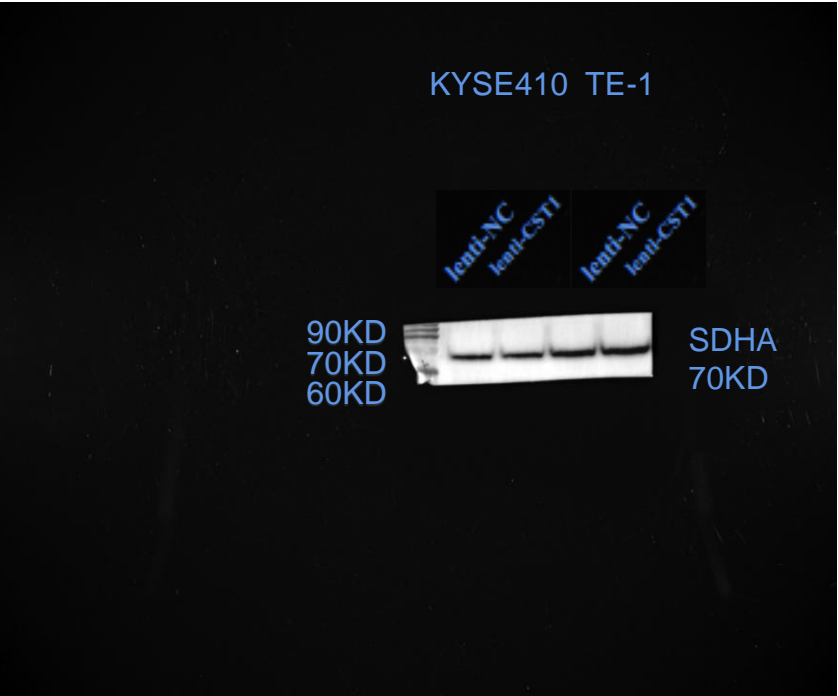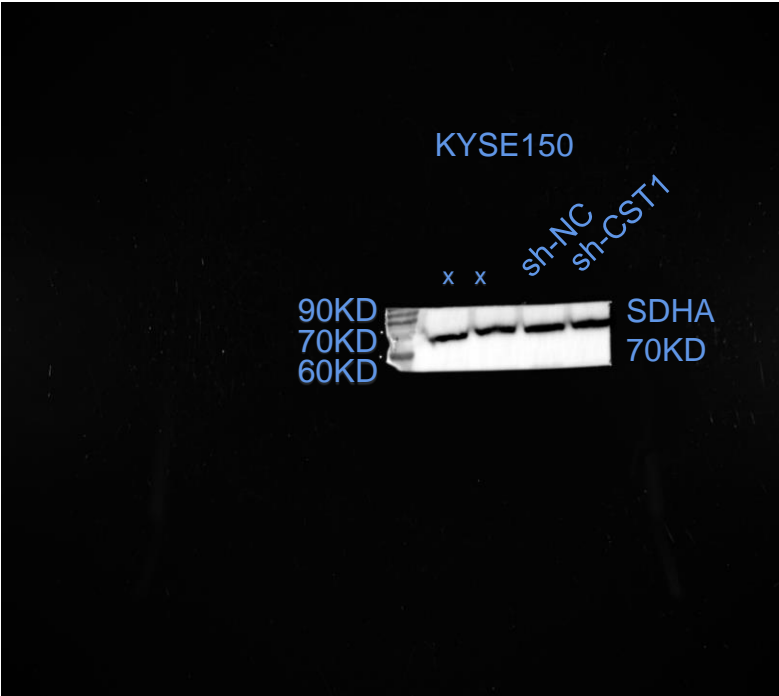

Fig 6D

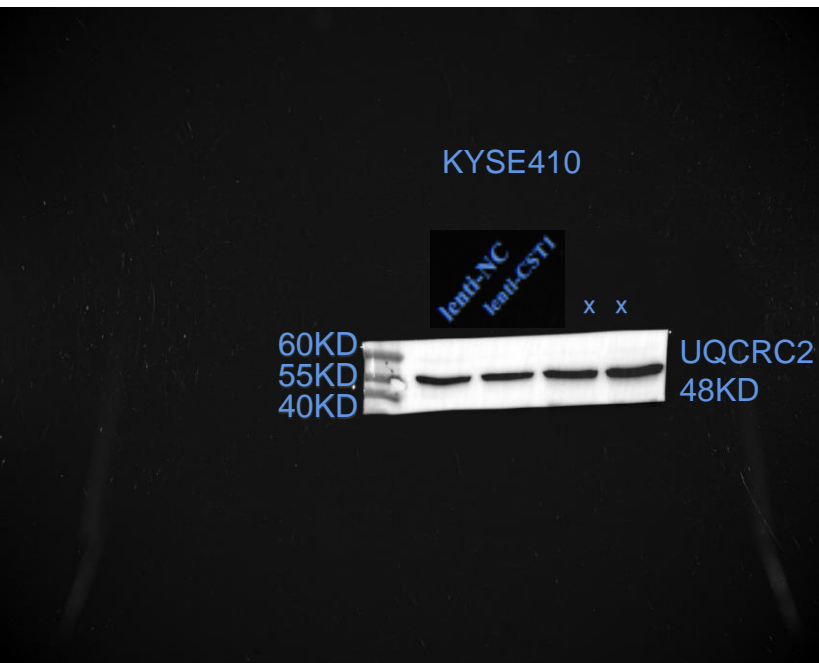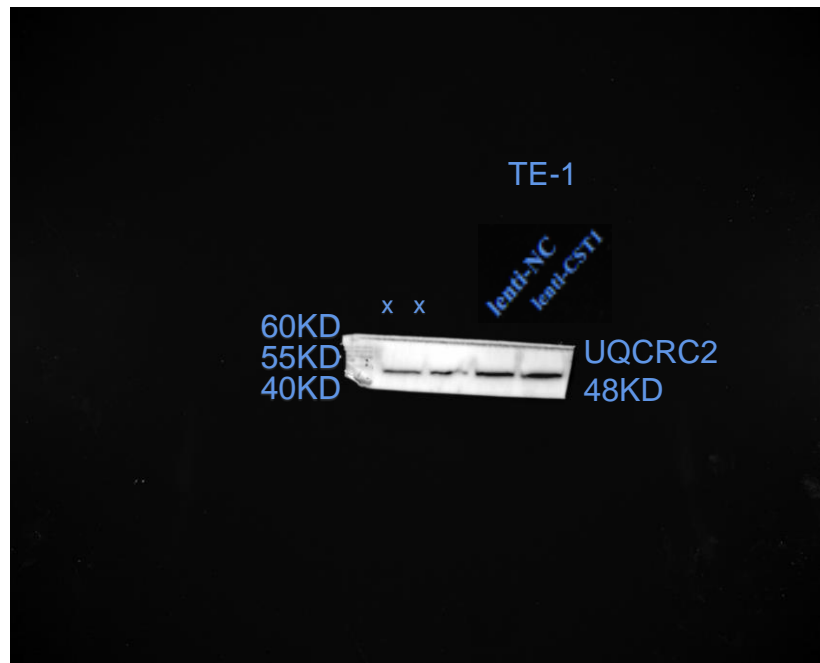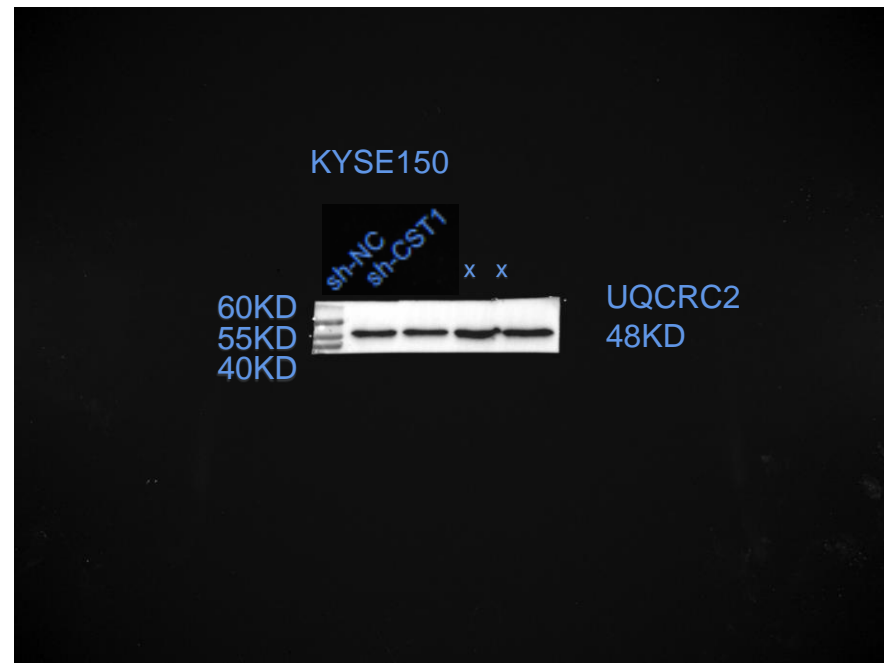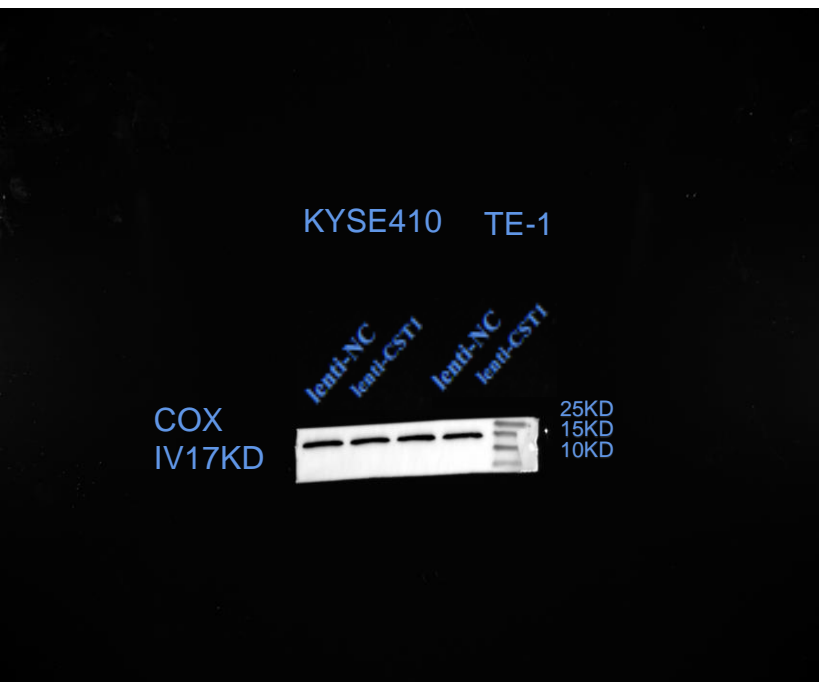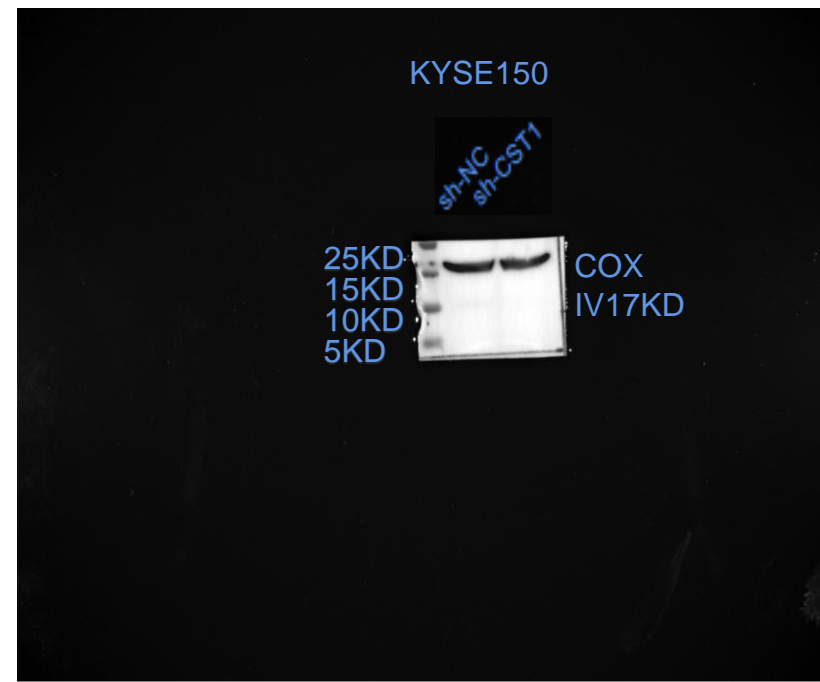

Fig 6D

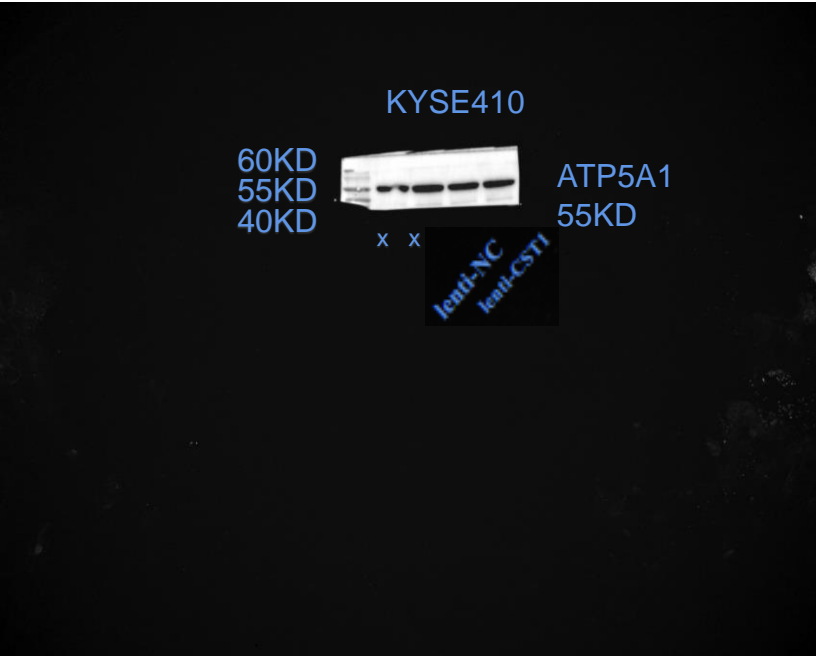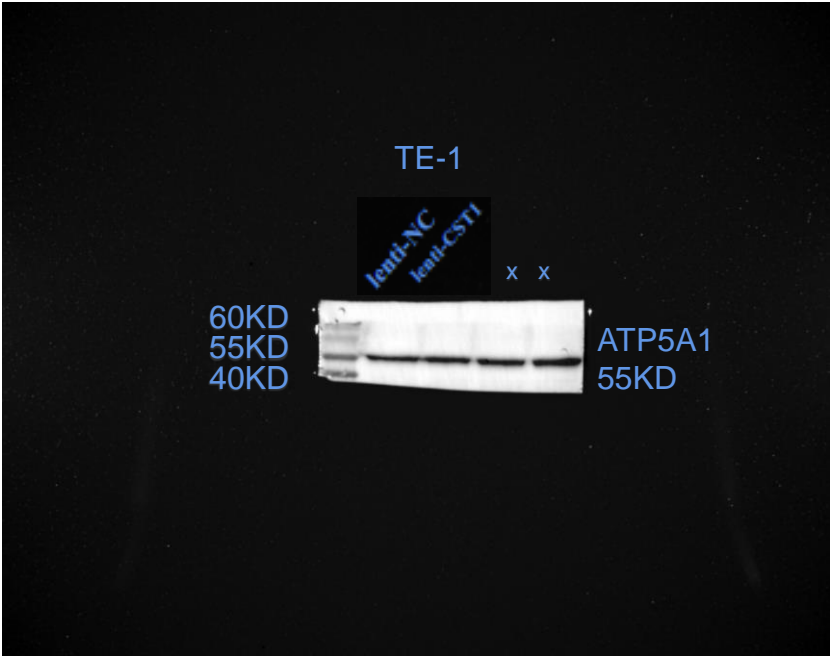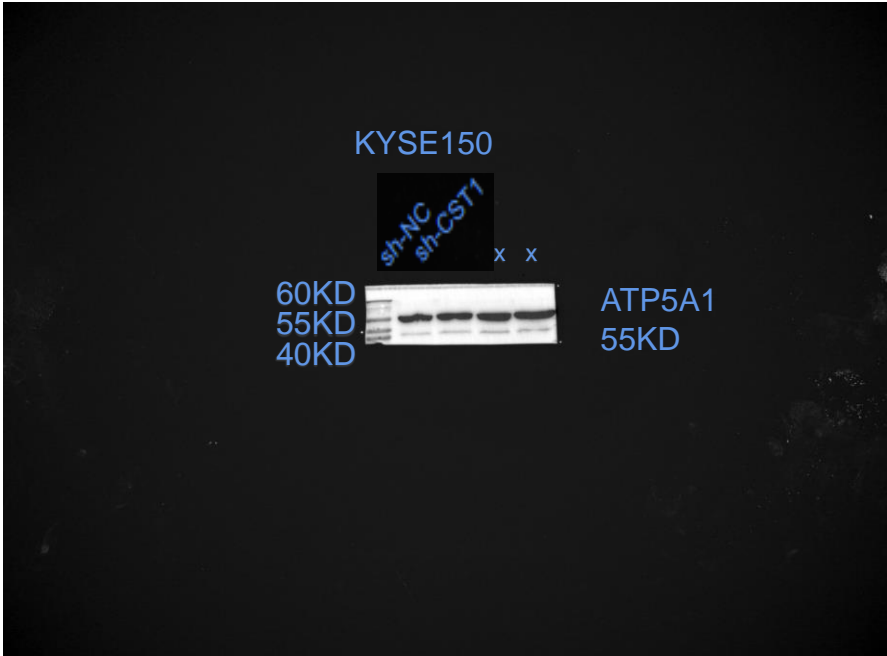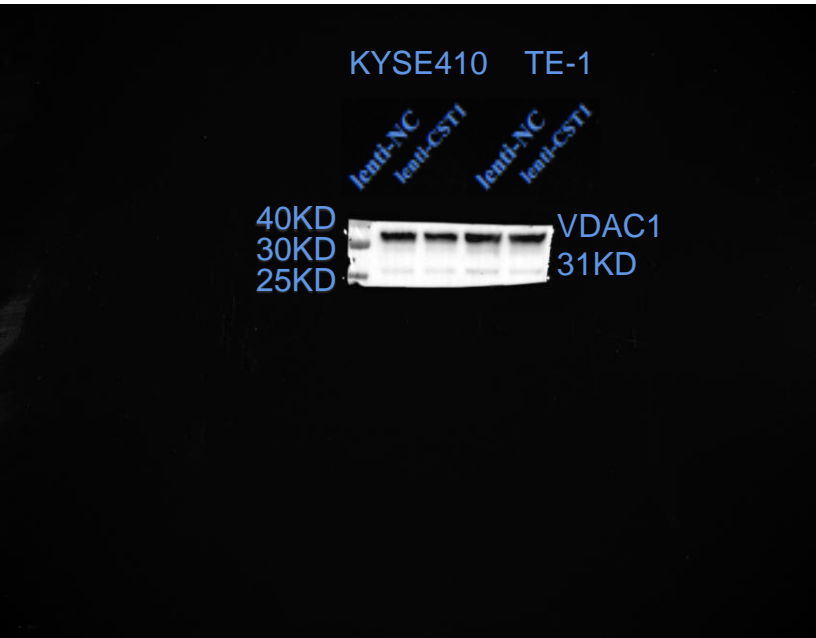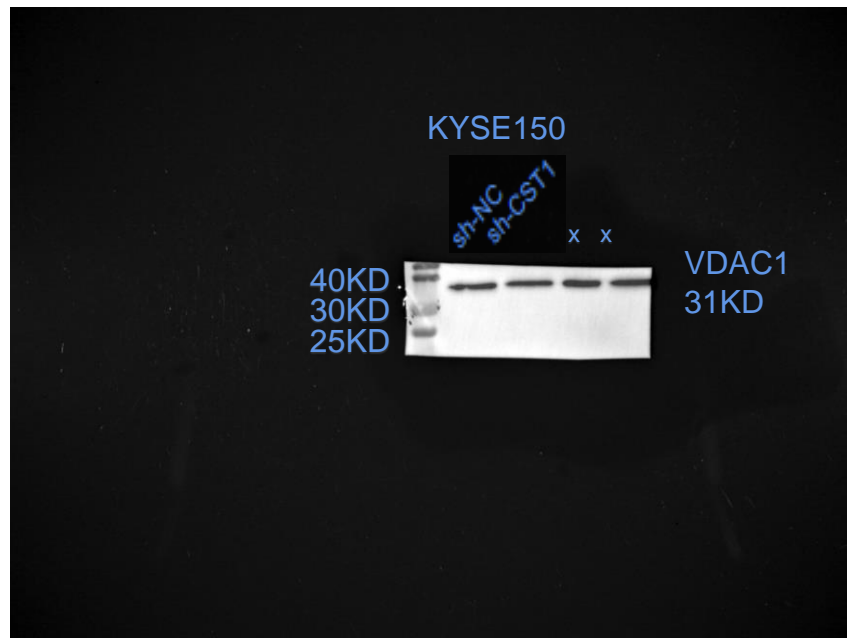

Fig 6D

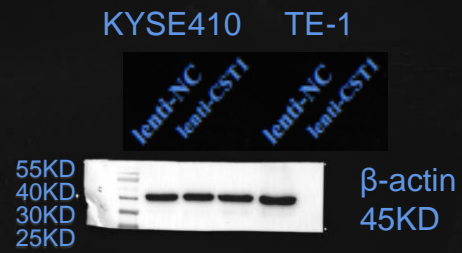

Fig 6E

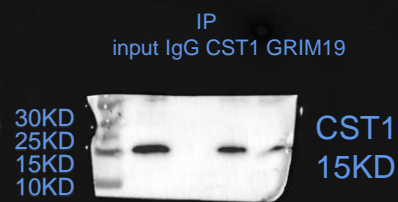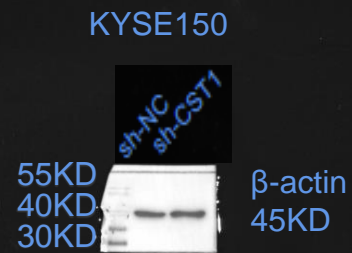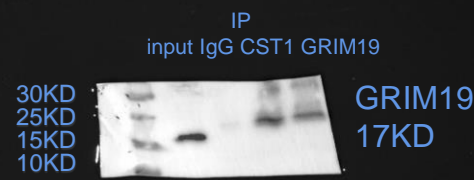

Fig 7A

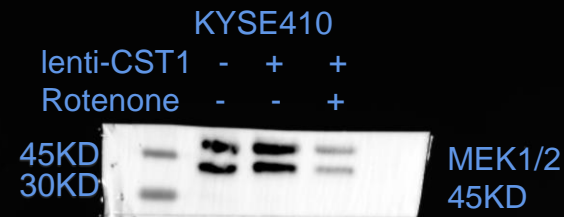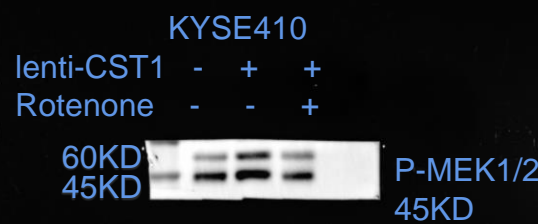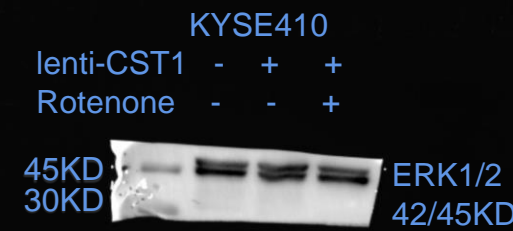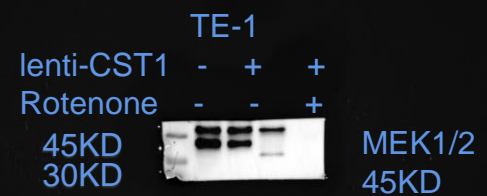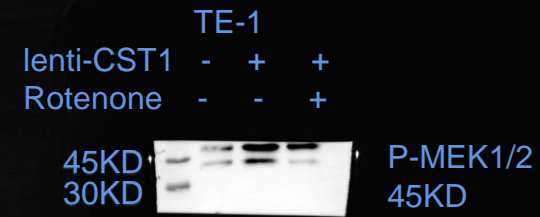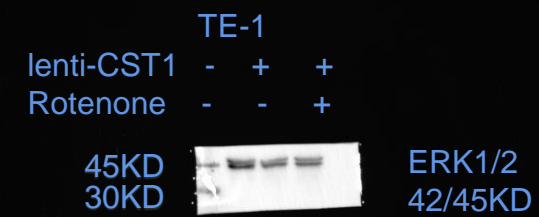

Fig 7A

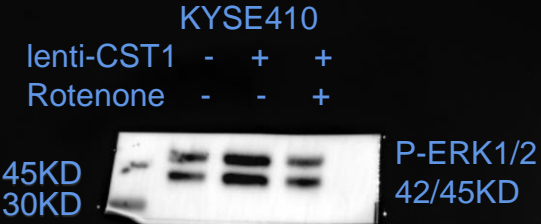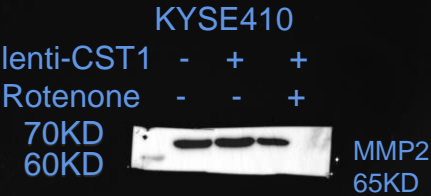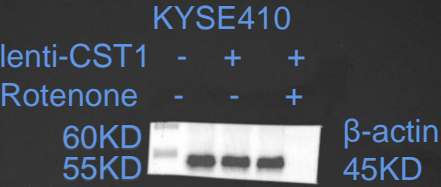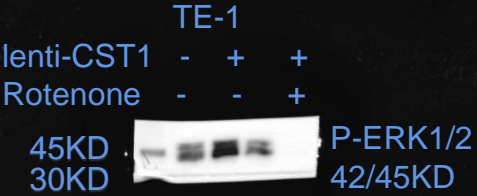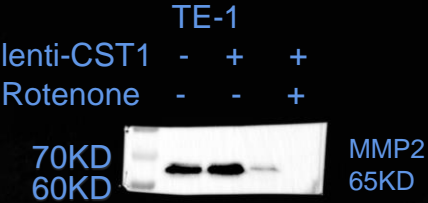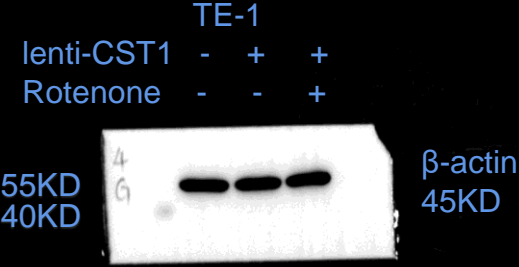

**Fig 9D**

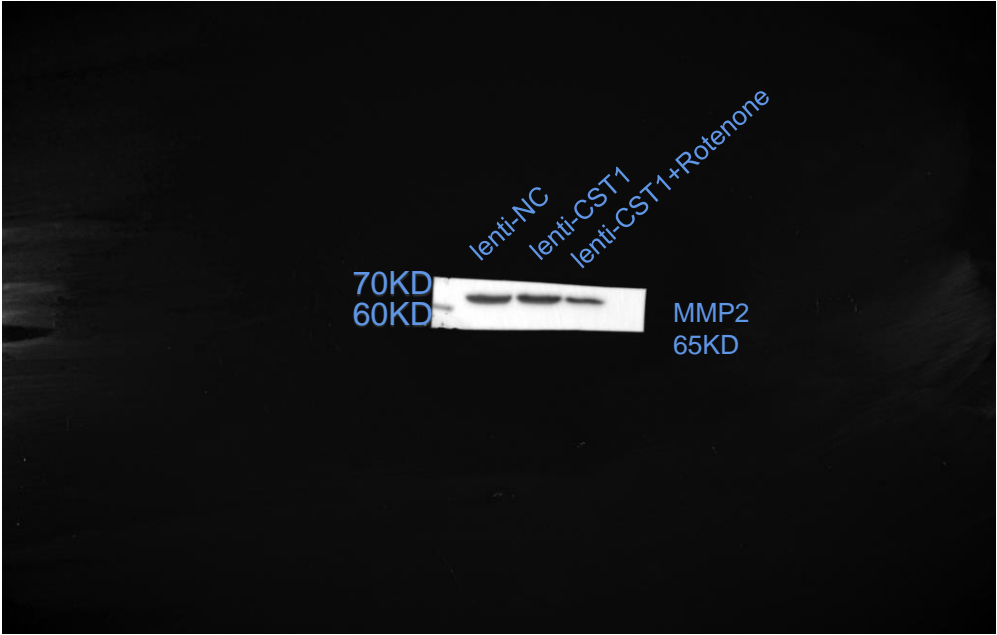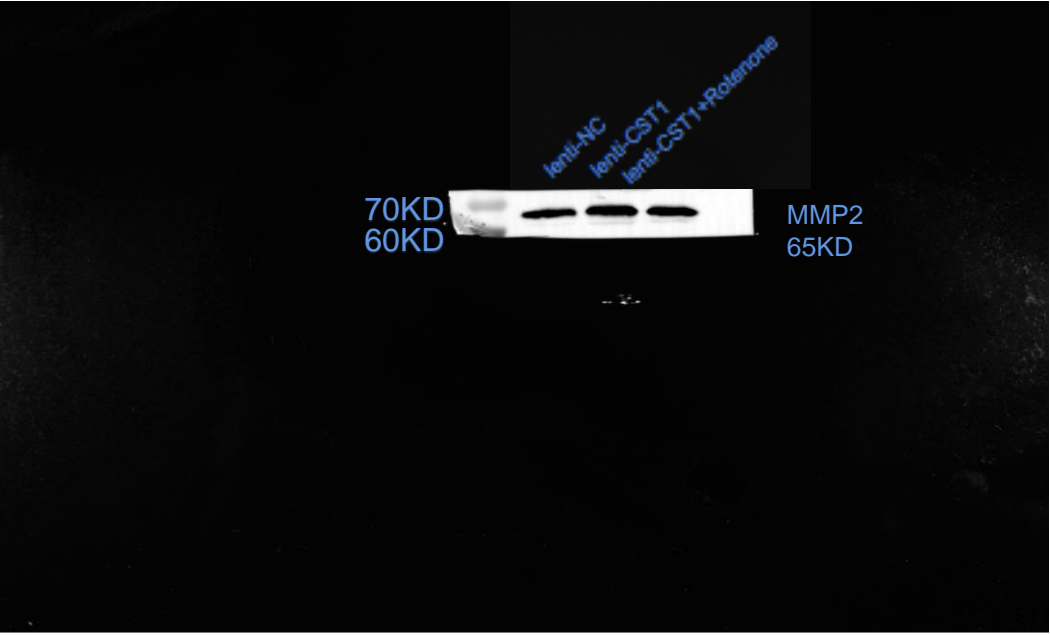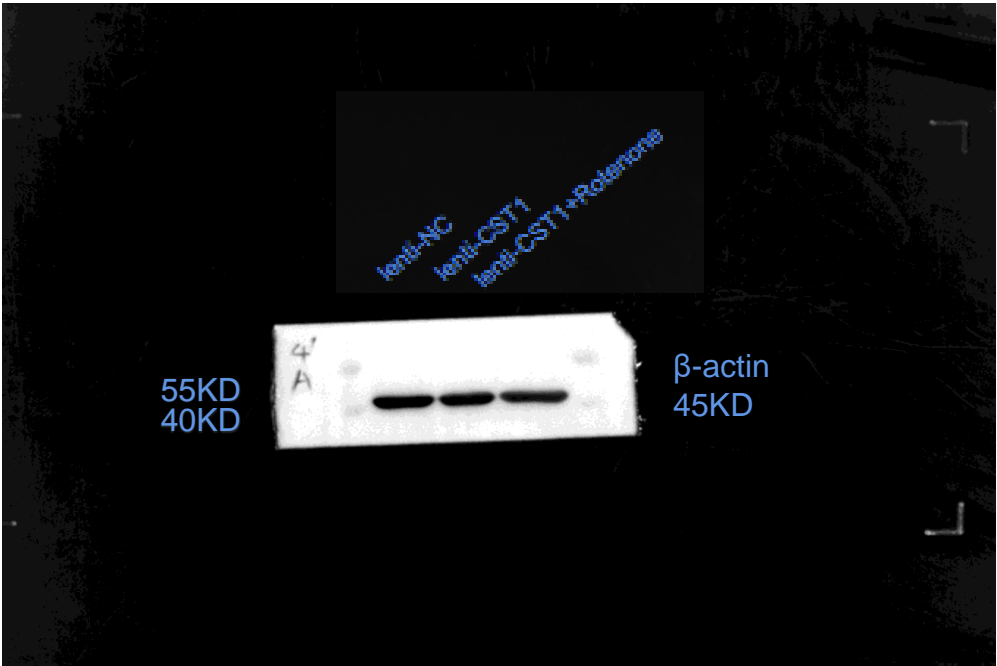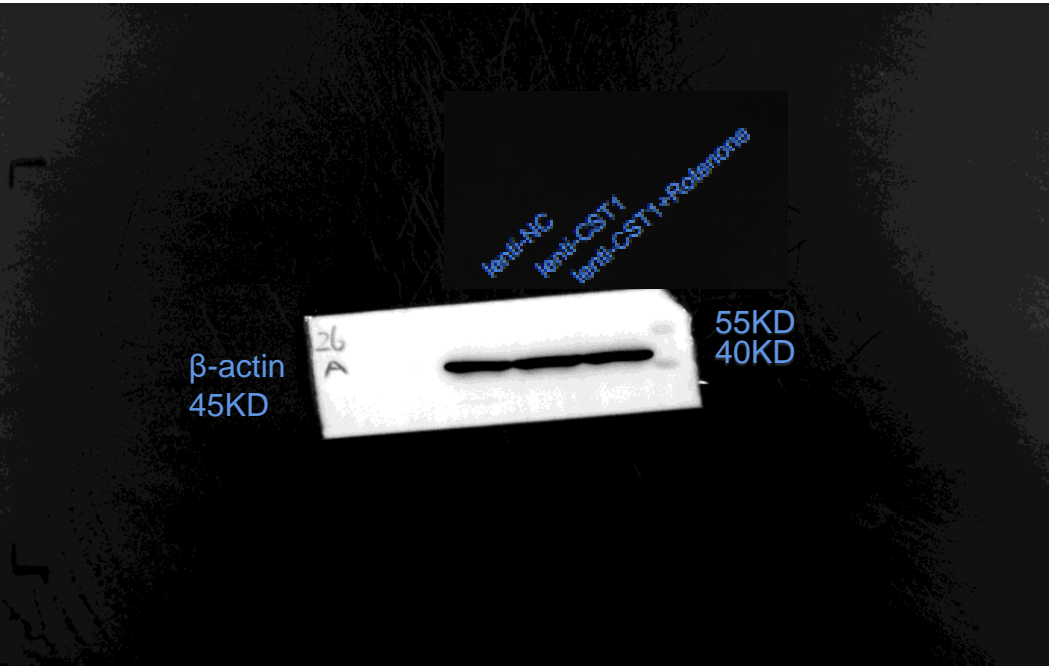

Supplement: Supplementary file 1 — Supplementary Information. [file 41598_2024_55544_MOESM1_ESM.pdf]
